# Polymorphic ROYalty: The 14th ROY Polymorph Discovered via High-Throughput Crystallization

**DOI:** 10.1021/jacs.4c17826

**Published:** 2025-03-25

**Authors:** Jake Weatherston, Michael R. Probert, Michael J. Hall

**Affiliations:** Chemistry, School of Natural and Environmental Sciences, Newcastle University, Newcastle upon Tyne NE1 7RU, U.K.

## Abstract

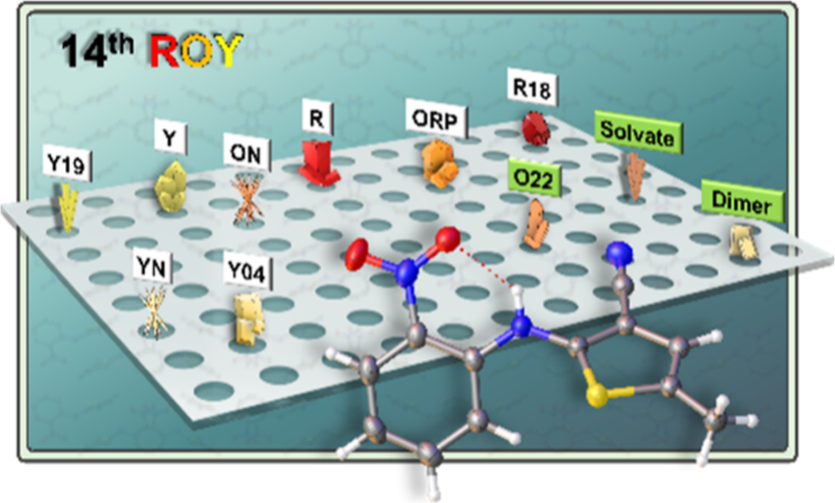

Polymorphism, when a substance can exist in more than one crystalline form yet return to the same liquid or solution phase, is characterized by differences in packing or molecular conformation. Polymorphs often exhibit differing physical properties, and are therefore particularly important in the development of materials and pharmaceuticals. However, gaining a thorough understanding of the solid-state landscape of a molecule requires exhaustive experimental screening of crystallization conditions, a particular challenge when using classical crystallization methods. We show that high-throughput Encapsulated Nanodroplet Crystallization (ENaCt) can enable the rapid and efficient exploration of the solid-state landscape of highly polymorphic molecules, through an in-depth study of 5-methyl-2-((2-nitrophenyl)amino)thiophene-3-carbonitrile (ROY), the most polymorphic small molecule known. An ENaCt screen encompassing 1536 individual crystallization experiments, spanning 320 unique conditions, resulted in direct access to single crystals, suitable for X-ray diffraction analysis, for all six of the known polymorphs accessible from solution (Y, R, YN, ON, ORP and R18). In addition, two polymorphs (Y04 and Y19) previously accessed only via melt and heteroseeded melt experiments, and a new polymorph of ROY (O22) were obtained. Furthermore, ENaCt screening resulted in the identification of the first ROY solvate (ROY· methyl anthranilate) and the first example of a ROY dimer, formed via in situ oxidation. ENaCt is thus shown to be an impactful tool for the experimental mapping of the solid-state landscape of highly polymorphic molecules and, through the discovery of a new polymorph O22, has ensured that tetradecamorphic ROY retains the record for the most polymorphic small molecule.

## Introduction

**Figure 1:**
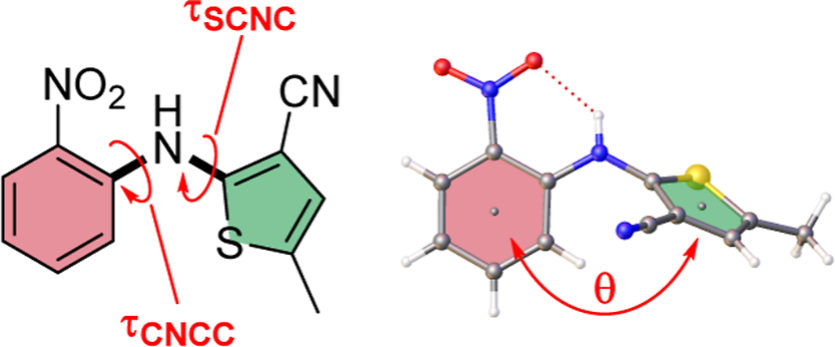
Molecular structure of 5-methyl-2-((2-nitrophenyl)amino)-3-thiophenecabonitrile (ROY). Key descriptors that relate to intramolecular conjugation in ROY highlighted, the phenyl-thiophene rotatable bonds and their associated torsion angles (τ_SCNC_ and τ_CNCC_) and the phenyl-thiophene mean plane angle (θ, illustrated with the Y polymorph).

Polymorphism occurs when a substance exists in multiple accessible crystalline forms. These differ only through the packing arrangement of atoms or molecules, or the molecular conformations within a crystal.^1−4^ Polymorphs can have different physical properties, such as stability, hygroscopicity, melting point, hardness, dissolution rate, and even interaction with light (giving rise to color).^5^ Polymorphism is of particular importance in the formulation of small molecule drugs, where different polymorphic forms can influence the bioavailability of the compound. Although polymorphism is relatively common, with approximately 4% of small organic molecules having reported polymorphs,^6^ there are few examples of highly polymorphic molecules known. These are defined as having nine or more characterized polymorphs such as aripiprazole,^7^ flufenamic acid,^8^ tolfenamic acid,^9^ galunisertib^10^ and nicotinamide.^11^ These highly polymorphic molecules often share common structural features, known as a “polymorphophore”, which may offer some explanation to their structural variety.^12,13^ The small organic molecule with the highest number of known polymorphs is the olanzapine precursor 5-methyl-2-((2-nitrophenyl)amino)thiophene-3-carbonitrile (Figure 1), also known as ROY due to the red, orange and yellow color of its crystals.^14^ ROY is the focus of the study presented herein.

Prior to this work, 13 polymorphs of ROY have been identified, 12 of which have reported structures obtained by single crystal X-ray diffraction: Y,^15^ ON,^15^ R,^15^ OP,^16^ YN,^16^ ORP,^16^ YT04,^17^ Y04,^17,18^ R05,^19,20^ PO13,^21^ R18,^22^ and Y19,^23^ named for their color [yellow (Y), orange (O), red (R), orange red (OR) and pumpkin orange (PO)], morphology [needle (N), plate (P/PL)] and later by year of discovery. RPL has also been identified, but full structural details have yet to be reported.^24,25^

Recent crystal structure prediction (CSP) studies indicate that all of the 13 previously known ROY polymorphs exist within 6.6 kJ/mol window of calculated lattice energies, from the most stable polymorph Y to the least stable Y19, with a further 59 predicted Z′ = 1 polymorphs lying within this energy range.^26^ Thus, despite the considerable research efforts expended by multiple research groups over >25 years in exploring the crystallization of ROY, CSP suggests that many new polymorphs may yet remain to be discovered.^25−27^

However, the discovery of new ROY polymorphs would require an exhaustive exploration of crystallization space in combination with methods to kinetically trap accessible higher energy forms, beyond the capacity of current approaches. In recent years we have developed the Encapsulated Nanodroplet Crystallization (ENaCt) technology to facilitate crystallization screening for small organic molecules.^22^ ENaCt takes advantage of the capabilities of modern liquid-handling robotics, to rapidly setup individual crystallization experiments using nanolitre scale droplets of organic solvent encapsulated within inert oils in a 96-well plate format. Oil encapsulation helps to mediate the rate of solvent loss from the droplets, allowing for slow concentration of the sample and ultimately the growth of single crystals suitable for direct X-ray diffraction (SC-XRD) analysis. ENaCt has, to date, been applied to the crystallization of a wide range of molecular systems including hypocrellin natural products,^28^ bicyclic triazolium salts,^29^ SARS-CoV-2 protease inhibitors,^30^ cannabidiol polymorphs,^31^ and flexible hydrogen-bonded organic frameworks (HOFs).^32^

High-throughput methods have previously been applied to the investigation of polymorphism in ROY, including high density polymer-induced heteronucleation (PIHn),^33^ and more classical robot-assisted thermal cycling from solution, coupled with RAMAN or PXRD analysis.^34^ However, to date such methods have not yielded new crystalline forms of ROY, despite considerable experimental space exploration. We hypothesized that the nanoscale crystallization droplets used in ENaCt would limit the total number of nucleation sites in each individual experimental well, in line with nanoscale confinement methods,^35−38^ increasing the chances of trapping new metastable (higher energy) polymorphs. Based on this we examined the solid-state landscape of ROY via ENaCt in the quest for new polymorphs, resulting in three new crystalline forms, including a new 14th polymorph.

## Results and Discussion

### High-Throughput Polymorph Screening by ENaCt

A high-throughput (HTP) ENaCt crystallization screen was implemented utilizing 32 solvents with and without water, in combination with 4 encapsulating oils and a “no oil” control. Water as an antisolvent was included as an experimental variable, as previous observations have shown that the metastable R18 polymorph grew preferentially from organic solvent/water mixtures.^22^ Since nucleation is a stochastic process, experimental replicates were also included (5 for each solvent/water/oil combination and 4 for each solvent/water/“no oil” control), resulting in 1536 individual ENaCt experiments covering 320 different experimental crystallization conditions, utilizing 16 96-well plates (see Supporting Information).

For each chosen solvent, a near saturated stock solution was prepared by portion wise addition of the minimum solvent required to dissolve ∼1 mg of ROY. Using an SPT Labtech Mosquito liquid handling robot, 100 nL droplets of these stock solutions were dispensed across 96-well LCP glass plates (100 μm spacer) into predispensed 300 nL oil droplets, or empty wells for “no oil” controls. In parallel, a second set of ENaCt experiments were prepared in which 100 nL ROY stock solutions and 25 nL of water were sequentially taken up by the liquid handling robot, prior to dispensing the mixed sample into 300 nL predispensed oil droplets. The 96-well plates were then sealed with a glass coverslip and monitored using cross-polarized and visible light microscopy over 7 days for the appearance of crystals, with outcomes ranked by apparent crystallinity.

**Figure 2:**
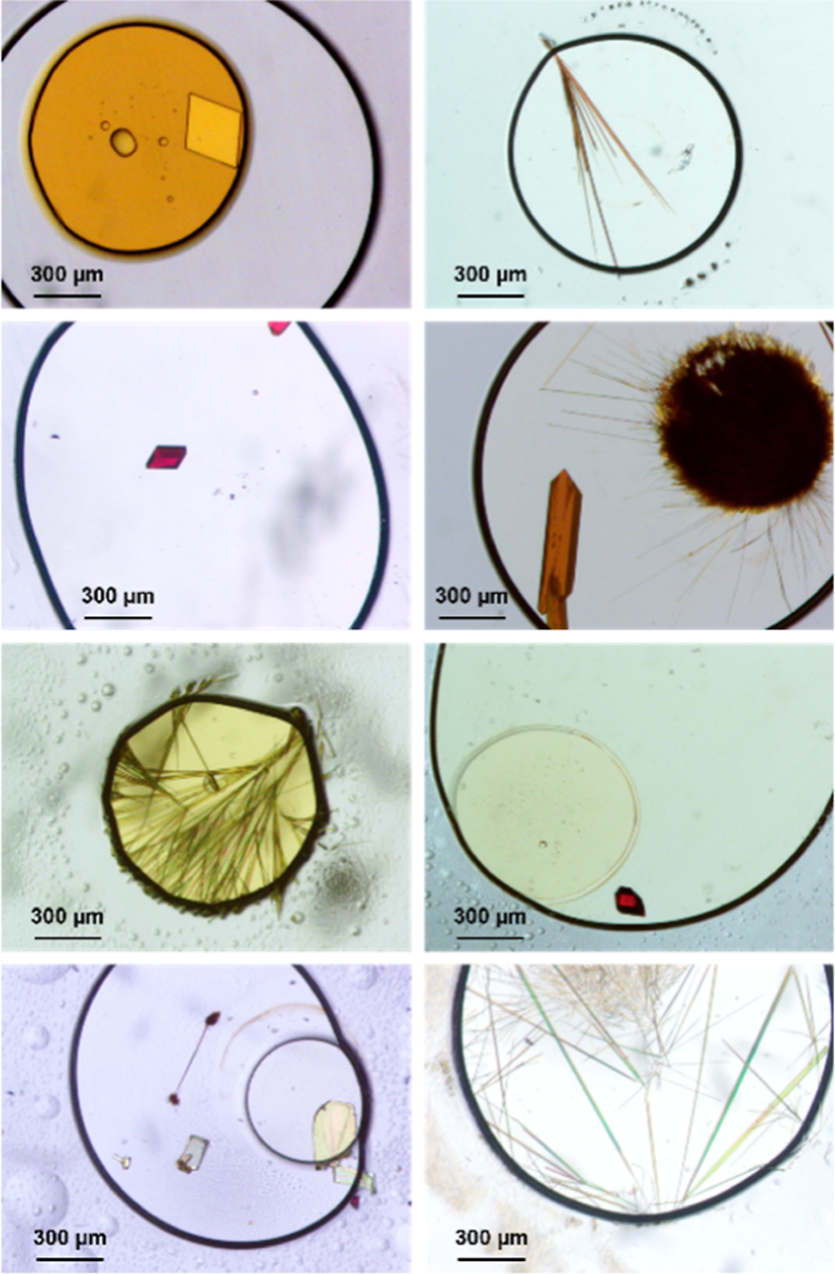
Optical microscopy of ROY crystals from ENaCt polymorph screen (top left to bottom right: Y, ON, R, ORP, YN, R18, Y04, Y19).

Due to the large number of crystallization experiments performed, initial analysis was undertaken using optical microscopy observation of color and morphology. This allowed the categorization of wells as containing crystalline material suitable for SC-XRD analysis and assignment of crystal form. Thus, 932 wells were identified as containing single crystals, with 1023 occurrences of specific crystal forms. Following which, a representative selection of crystals was examined across different experimental conditions by SC-XRD, the crystal form assignment was validated by unit cell measurements for 310 wells (∼30%), with full structure solution undertaken for each different form detected (see Supporting Information). This resulted in the identification by SC-XRD of 11 ROY related crystals, including nine different ROY polymorphs consisting of eight known forms (Figure 2) and one previously unreported polymorph (O22), a ROY dimer and a ROY solvate.

Of the eight known polymorphs obtained from ENaCt screening, five had been previously observed from classical solution-phase crystallization (Y, ON, R, ORP, YN), along with one that was previously discovered using ENaCt (R18). Interestingly, two ROY polymorphs were also obtained that had previously only been accessed via melt microdroplet crystallization (Y04) or heteroseeded melt crystallization (Y19).^17,23^ The observation of “melt-only” polymorphs supports the proposal that metastable forms, beyond those accessible to classical solution phase approaches, can be accessed through ENaCt methods.

Only four of the known polymorphs eluded our screening, none of which are known to crystallize from solution, two of which were discovered from melts (OP, PO13), one from cross-nucleated melts (R05), and one formed via sublimation onto the (010) surface of succinic acid crystals (RPL) for which no SC-XRD data has been previously reported, but for which structures have been proposed.^24,25^

### 14th ROY Polymorph O22

**Figure 3:**
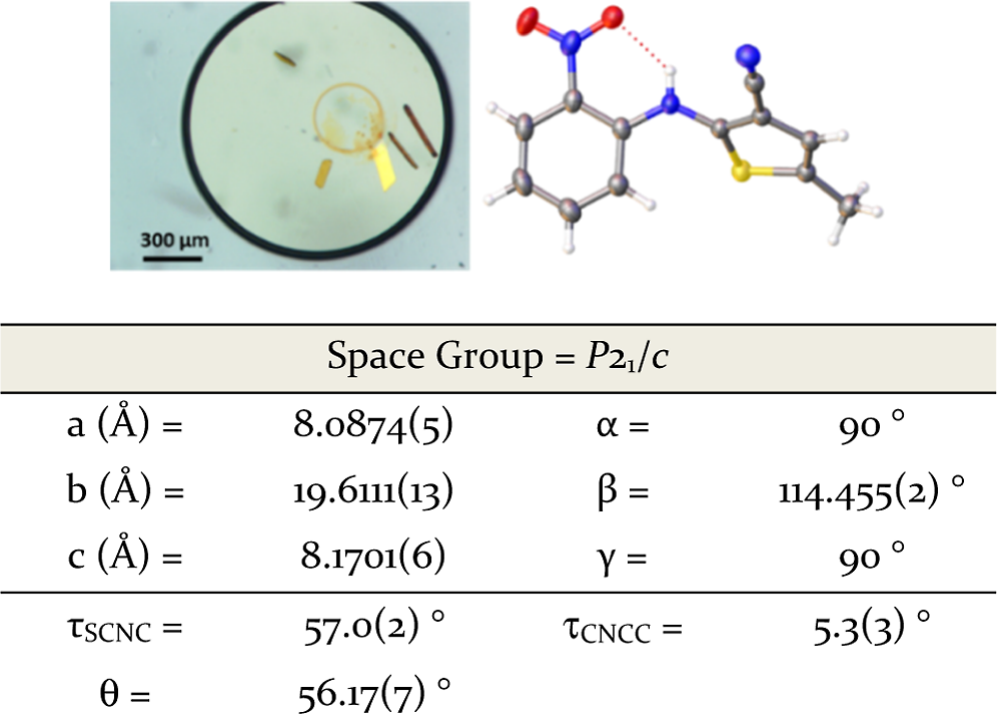
Optical microscopy of O22 crystals observed from ENaCt polymorph screen (50 mg/mL ROY in DMSO/mineral oil), crystal structure of O22 (anisotropic displacement parameters shown at 50%), and selected crystal structure parameters.

Excitingly, a new orange polymorph of ROY was discovered from a solution of ROY in dimethyl sulfoxide (50 mg/mL ROY in DMSO/mineral oil), herein named O22 (orange 2022). O22 was only observed in a single experimental well, where five discrete orange crystals with a block morphology were formed, all of which gave the same unit cell dimensions determined from SC-XRD. The complete structure of O22 was established, showing that it had crystallized in the monoclinic *P*2_1_/*c* space group, with Z’ = 1 (Figure 3).

Like many of the ROY polymorphs, O22 shows an intramolecular H-bond between the arylnitro and diarylamine groups, which flattens the molecule as seen in the small τ_CNCC_ torsion angle (−5.3(3)°). O22 also contains head-to-tail dimers, in which two molecules of ROY are related via an inversion center, resulting in intermolecular nitrile-to-nitrile π–π interactions and diarylamine to nitrile hydrogen bonds. Furthermore, both phenyl–phenyl and thiophene–thiophene π–π stacking interactions can be observed in the [001] and [100] directions respectively (see Supporting Information).

In ROY polymorphs, the observed crystal color often correlates with the extent of conjugation between the two ring systems. The SCNS torsional angle (τ_SCNC_) and the mean plane angle (θ) are often used to estimate the extent of conjugation, and thus to map to polymorph color.^14,39−42^ In O22, τ_CNCS_ = 57.0(2)° and θ = 56.17(7)°, and thus are consistent with the other known orange polymorphs (see Supporting Information).

Interestingly, the structure of O22 appeared to closely match that of a previously predicated polymorph, disclosed by the group of Beran (reported as “Structure Rank no. 24”).^26^ The packing similarity between Structure Rank no. 24 and O22 was assessed using the Crystal Packing Similarity feature within Mercury 4.0, based on the COMPACK geometry analyses of molecular clusters.^43,44^ A 15-molecule cluster of both structures was calculated using the default tolerance values (20% for distances between overlaid molecules and 20 degrees for angles between overlaid molecules). Successful overlay of all 15 molecules with a root-mean-square deviation (RMSD) of 0.137 Å, demonstrated an excellent match between the predicted and experimental structures, supporting the assignment of CSP Structure Rank no. 24 as polymorph O22 (further supported by related CrystalCMP calculations, see Supporting Information).^45^ Mapping onto Beran’s original CSP study, this suggests that O22 has a lattice energy 4.4 kJ/mol higher than polymorph Y.^26^ Thus, O22 sits within the window of lattice energies defined by the other 13 experimentally reported forms, slightly above ORP (*E*_rel_ = 4.3 kJ/mol) and below R18 (*E*_rel_ = 4.9 kJ/mol) (see Supporting Information). It should be noted that early CSP work by Vasileiadis, which successfully predicted the subsequently discovered ROY polymorphs Y04 and Y19, also predicted O22, being a close match to structure no. 134 (15 molecule overlay RMSD = 0.145 Å).^27^

With the discovery of O22 as the 14th reported polymorph, ROY becomes the first known tetradecamorphic system and retains the record for the most polymorphic small molecule known.

### ROY Dimer

**Figure 4:**
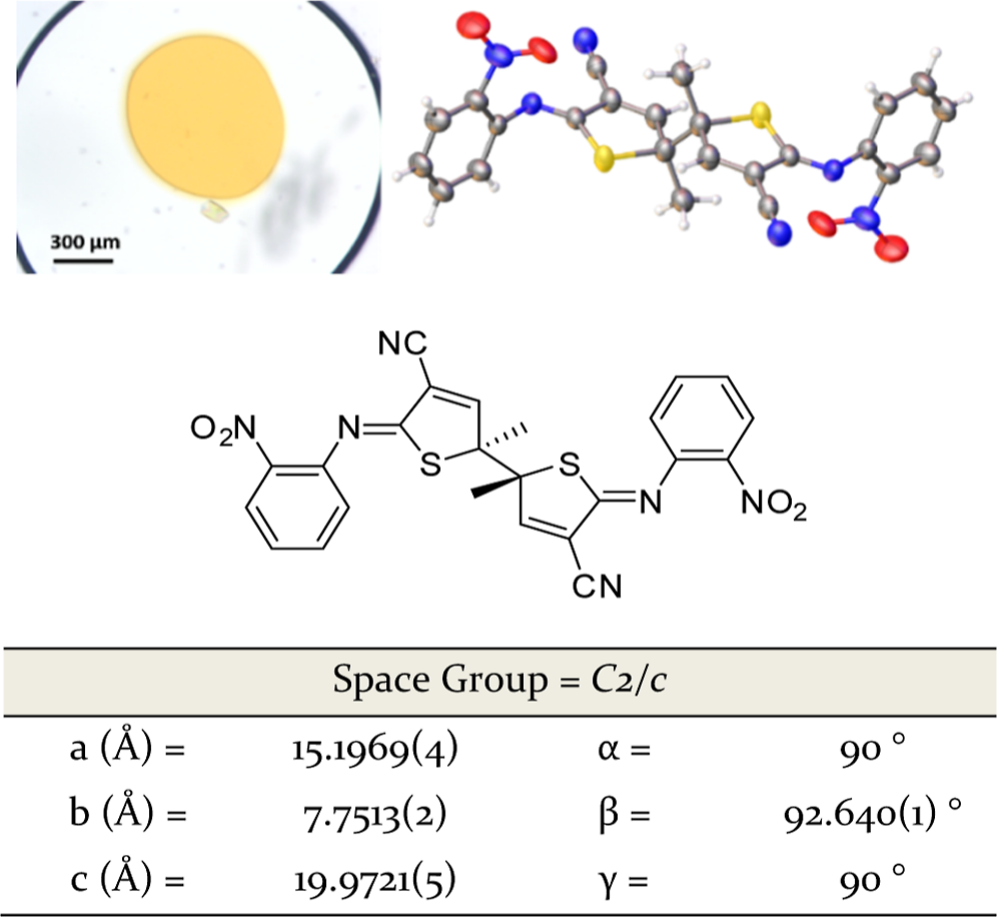
Optical microscopy of ROY dimer crystal observed from ENaCt polymorph screen (14.3 mg/L ROY in 2-nitroethanol/mineral oil), crystal structure of ROY dimer (anisotropic displacement parameters shown at 50%, disordered nitro groups displayed as the major disorder component for clarity), molecular structure and selected crystal structure parameters.

The ENaCt screen also revealed a single experimental well containing a yellow plate-like crystal (14 mg/mL ROY in 2-nitroethanol/mineral oil), with no other crystals observed from the use of 2-nitroethanol as the solvent. Analysis by SC-XRD revealed that the crystal did not contain ROY itself, but the meso-diastereomer of a molecule formed via an oxidative dimerization of ROY (Figure 4).

This ROY dimer had crystallized in the monoclinic *C*2/*c* space group with a Z′ = 0.5, with the molecule containing an inversion center. The ROY dimer shows significant structural changes from the known ROY polymorphs. In particular, the transformation of a bridging amine to an imine results in the extended planarization of the newly formed sulfur heterocycle (τ_SCNC_ = 7.8(6)°), with concurrent loss of the nitro to amine intramolecular S(6) H-bond (τ_CNCC_ = 75.6(5)°).

We postulated that the observed ROY dimer was likely formed during the crystallization experiment, as no evidence of the dimer can be observed by NMR or HRMS of the initial ROY samples (see Supporting Information). We propose that due to poor crystallization of ROY from 2-nitroethanol, the sample remains in solution for a sufficiently long time for an oxidative coupling to occur, likely mediated by atmospheric oxygen. The highly crystalline ROY dimer then preferentially crystallizes from solution.

### Methyl Anthranilate Solvate of ROY

**Figure 5:**
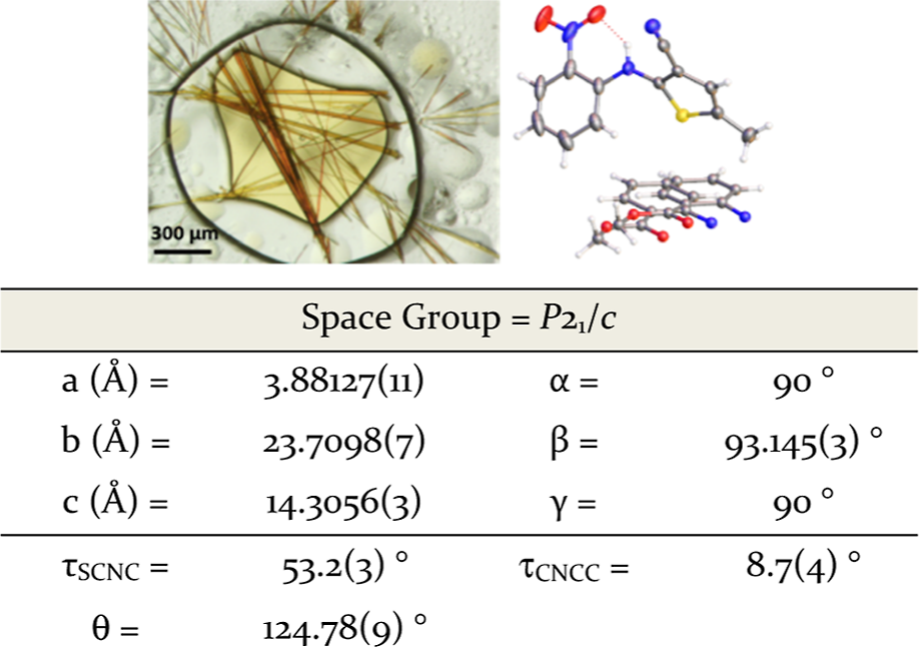
Optical microscopy of ROY/methyl anthranilate observed from seeded ENaCt experiments [50 mg/mL ROY in methyl anthranilate/mineral oil(seeded)], crystal structure of ROY/methyl anthranilate (anisotropic displacement parameters shown at 50%, disordered solvent modeled over two positions as shown), and selected crystal structure parameters.

Additionally, during the ENaCt polymorph screen, orange needle-like crystals were observed to grow from methyl anthranilate (50 mg/mL of ROY in methyl anthranilate) in all four encapsulating oils, that did not match known forms of ROY through either morphology/color, or via unit cell analysis. Only weak diffraction was obtained, however this was sufficient to obtain both space group (*P*2_1_/*c*) and unit cell parameters. Interestingly, the new form showed similarities to Y19, but with an elongation of the *c* axis (12.5 to 14.3 Å). To obtain higher quality crystals a follow up seeded ENaCt experiment was undertaken in which the original needle-like crystals were harvested, pooled and crushed to generate a microcrystalline seed stock. Seed material was then suspended in mineral oil, and this “seeded” mineral oil was used as the encapsulating material for the next set of ENaCt experiments (96 well plate, 50 mg/mL ROY in methyl anthranilate, mineral oil).^31^ This resulted in rapid growth of the target crystal form, only 1 day elapsing before all 96 wells contained orange needle-like crystals, with significantly improved crystal volume and quality, resulting in successful structure solution by SC-XRD (Figure 5).

SC-XRD showed that ROY had crystallized with methyl anthranilate to form a nonstoichiometric channel solvate, with a solvent occupancy of 0.220(3) per ROY molecule. Within the ROY/methyl anthranilate solvate, ROY showed a packing motif with high similarity to Y19 (supported by similarity comparison score of 3.06 using CrystalCMP, see Supporting Information).^45^

The major structural difference from Y19 was the presence of an increased spacing between adjacent ROY molecules in the crystallographic [100] direction, creating a void. Channels of residual electron density running down the [001] direction in this void space were modeled as disordered methyl anthranilate extending in a continuum down the channels (see Supporting Information). Although the occurrence of solvates of small organic molecules is common, ROY/methyl anthranilate is the first reported solvate known for the highly studied ROY system.

### Impact of ENaCt on Polymorph Propensity

**Figure 6:**
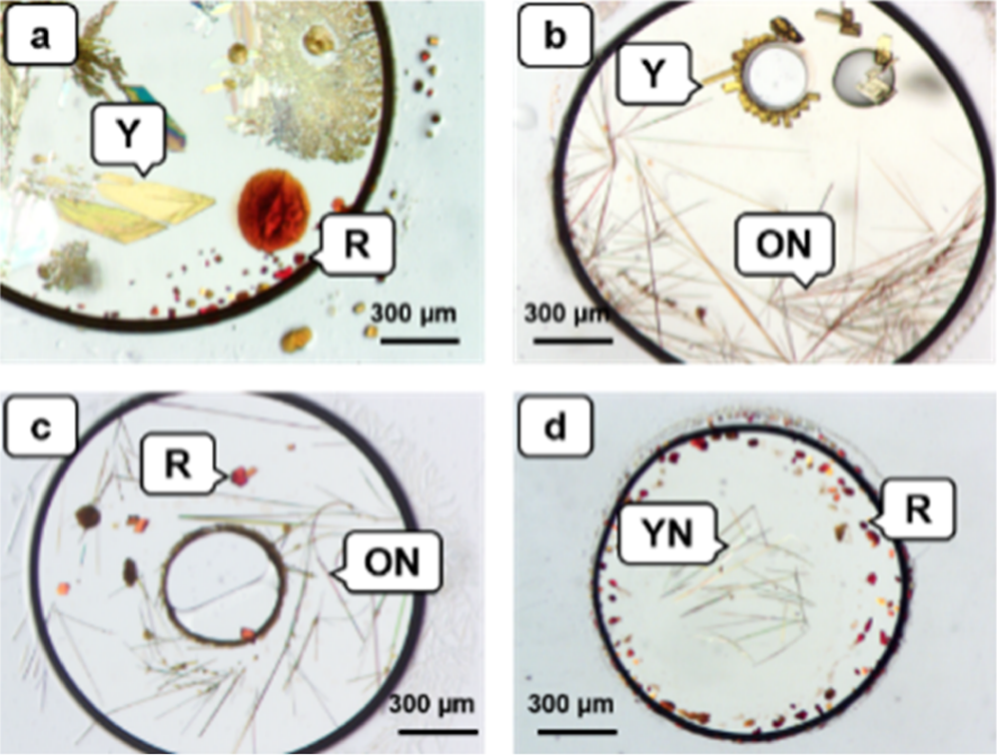
Optical microscopy of concomitant crystallization of ROY polymorphs, from ENaCt screen. (a) Y and R, (b) Y and ON, (c) R and ON, (d) R and YN.

To better understand the ability of ENaCt to access different polymorphs, the propensity of polymorph occurrence was examined across the whole polymorph screen. From 1536 individual crystallization experiments, 932 wells contained 1023 occurrences of identifiable ROY crystal forms, with 843 containing a single crystal form, and 89 wells exhibiting concomitant crystallization of more than one crystal form (87 wells containing two different polymorphs, and 2 wells containing three different polymorphs) (Figure 6, and Supporting Information).

Of the 1023 wells containing of identifiable ROY single crystal forms, 1016 were ROY polymorphs with 7 occurrences of the ROY dimer and ROY solvate forms. ON was the most commonly observed with 404 wells containing ON crystals, 26.3% of the total. The most thermodynamically stable Y polymorph was second (369, 24.0%), followed by YN (114, 7.4%) and R (93, 6.1%) which together made up the majority of the polymorphs observed, R18 (17, 1.1%), Y04 (9, 0.6%), Y19 (5, 0.3%), ORP (3, 0.2%) and O22 (1, 0.1%) being somewhat rarer. ON and YN are kinetically favored polymorphs, while the thermodynamically most stable Y is typically formed under equilibrium conditions.^16^ Thus, the observation of ON and YN as major polymorphs in ENaCt further supports the hypothesis that this approach can successful trap high relative energy kinetic polymorphs. The ENaCt experiments which included water as an antisolvent showed an increase in the presence of higher energy metastable forms, YN increasing by 59% (44 occurrences in 768 ENaCt experiments without water → 70 in 768 ENaCt experiments with water), R18 by 83% (6 → 11), while ORP (0 → 3) and Y19 (0 → 5), the highest energy polymorph, were only observed when water was present.^36^ In these cases it is likely that the presence of an antisolvent in the ENaCt droplet, speeds crystallization and aids in the kinetic trapping of metastable polymorphs, allowing access to “melt-only” form such as Y19.^46^

## Conclusions

Our investigation of the ROY polymorph landscape has spanned 1536 individual crystallization experiments, including 320 unique crystallization conditions. This has resulted in access to eight known ROY polymorphs (Y, ON, YN, R, ORP, Y04, Y19 and R18), including two metastable crystal forms that were previously considered inaccessible to solution phase crystallization (Y04 and Y19). ENaCt screening has also revealed a novel ROY solvate, an unexpected ROY dimer, and the 14th discovered polymorph (O22). Whether the highly polymorphic nature of ROY is due to an intrinsically privileged structure, or is a function of the effort applied to it is study, remains to be seen. High-throughput encapsulated nanodroplet crystallization (ENaCt) is thus shown to be a powerful tool for exploring the solid-state landscape of a small molecule, capable of accessing thermodynamically stable forms as well the formation and trapping of high energy metastable polymorphs, including “melt-only” forms. ENaCt outputs thus bridge both classical thermodynamically controlled crystallizations, and those methods more suited to finding kinetic forms. The observed analogy between the kinetic trapping of metastable polymorphs by ENaCt and by melt crystallization will be the subject of further studies to better understand the mechanisms of crystal formation in ENaCt.

## Supporting Information Available

Experimental information, including ENaCt and classical crystallization procedures and outcomes, crystallographic data tables, packing similarity analysis, and further structural discussion of new ROY forms (PDF)
