# Supplementary material for: Polymorphic ROYalty: The 14th ROY Polymorph Discovered via High-Throughput Crystallization
Source: J Am Chem Soc. 2025 Mar 25;147(14):11949–54. doi: 10.1021/jacs.4c17826 (PMC11987018; doi:10.1021/jacs.4c17826)
Supplement: Supplementary file 1 — ja4c17826_si_001.pdf [file ja4c17826_si_001.pdf]

Polymorphic ROYalty: The 14th ROY Polymorph Discovered via High-Throughput Crystallization

Jake Weatherston,[a] Michael R. Probert*[a] and Michael J. Hall*[a]

[a]Chemistry, School of Natural and Environmental Sciences, Newcastle University, Newcastle upon Tyne, UK.

Supporting Information

**S1. General Experimental Information**	S3
**S2. ENaCt Oils**	S3
**S3. ROY Stock Solutions**	S4
**S4. ENaCt Dispensing Protocols**	S5
**S5. ENaCt Polymorph Screen**	S10
**S6. ROY Crystal Forms Observed by Crystallisation Condition**	S13
**S7. Improved Crystallisation of ROY·methyl anthranilate solvate by Seeded ENaCt**	S17
**S8. Comparison of O22 with the CSP Structure Rank #24**	S17
**S9. Packing Similarity Analysis of Monomeric ROY Crystal Forms**	S18
**S10. O22 Crystal Structure Analysis**	S19
**S11. ROY·methyl anthranilate solvate Crystal Structure Analysis**	S23
**S12. ROY Dimer Crystal Structure Analysis**	S27
**S13. Purity Analysis of ROY using in ENaCt Experiments**	S30
**S14. Beran CSP Ranking of ROY Polymorphs including O22**	S32
**S15. Torsional and Mean Plane Angles of ROY forms**	S33
**S16. Crystallographic Data Tables for Obtained ROY Forms**	S34
**S17. References**	S37

S1. General Experimental Information

ENaCt experiments were completed using an STP LabTech mosquito® Xtal3 liquid handling robot.

Encapsulating oils and crystallisation solutions are dispensed from 'parent plates', which contain the bulk solutions and oils, into 'crystallisation plates' in which the crystallisation experiment is carried out. *'Parent plate' used for oils:* Greiner PS Microplate F-bottom non-sterile. Greiner item no. 655101. *'Parent plate' used for crystallisation solutions:* Thermo Scientific™ WebSeal Plate+ 96-Well Glass-Coated v-bottomed Microplate. *Crystallisation plate:* Swissci Modular Glass Base + 100µm 5.5mm Double Sided Adhesive - Pack 10 - Code: LCP-BASEG-100 sealed with Swissci Modular Cover Glass 73x109mm NO 1.5 - Pack 10 - Code: LCP-G175.

Visualisation of experiments was carried out with a Nikon SMZ1000 microscope fitted with a cross polariser, with photographs taken using a GXCAM-U3-5 5.1MP camera.

Crystals were identified using cross-polarised optical microscopy and harvested from the crystallisation plates using a tungsten carbide scribe to remove the glass coverslip. Crystals were then extracted under Fomblin YR-1800 oil with a Mitegen Kapton loop.

SCXRD data for forms O22, ON, R, Y, Y04, YN were collected using a Bruker D8 Venture, Photon 2, dual Incoatec IµS (Cu $K\alpha$, λ = 1.54184 Å) equipped with an Oxford Cryosystems Cryostream open-flow cooling device and maintained at 150 K.[S1] Data were reduced using Apex3, using SADABS absorption corrections.[S2]

SCXRD data for forms Y19, ORP, R18 were collected using a Rigaku Oxford Diffraction Synergy-S diffractometer (Cu $K\alpha$, λ = 1.54184 Å) with a hybrid pixel array detector. Samples were cooled to 150 K and temperature maintained using Oxford Cryosystems Cryostreams open-flow cooling device.[S1] Data were reduced using *CrysAlis PRO* with SCALE 3 ABSPACK correction implemented.[S3]

All structure solution and refinement were completed using the SHELX suite of programs via the Olex2 interface.[S4-6]

S2. ENaCt Oils

**Oils**	
PDMSO	Poly(dimethylsiloxane); CAS: 63148-62-9; supplier: Sigma Aldrich
FC-40	Fluorinert FC-40; CAS: 51142-49-5; supplier: Fluorochem
FY	Fomblin YR-1800; CAS: 69991-67-9; supplier: Alfa Aesar
MO	Mineral oil; CAS: 8042-47-5; supplier: Sigma Aldrich

Table S1: Oils used in ENaCt experiments.

S3. ROY Stock Solutions

An initial qualitative solubility test was used to determine suitable crystallisation solvents/antisolvents, following which 32 solvents and 1 antisolvent were selected. Stock solutions were prepared by weighing approximately 1 mg of ROY into 64 different 2 mL screw top glass vials. Solvent was added in 10 µL portions until the sample was fully dissolved, as confirmed by optical microscopy.

	Solvent	Mass of ROY (mg)	Approximate Concentration (mg mL^{-1})
1	**Formamide**	1.0	10.0
2	**1,4-Dioxane**	1.0	50.0
3	**4-Methyl-2-pentanone**	1.0	25.0
4	**Benzonitrile**	1.0	50.0
5	**2,2,2-Trifluoroethanol**	1.0	25.0
6	**2-Fluoroanisole**	1.0	50.0
7	***n*-Butyl acetate**	1.0	25.0
8	**Dimethyl carbonate**	1.0	25.0
9	**1,2-Dichloroethane**	1.0	50.0
10	**1-Formylpyrrolidine**	1.0	50.0
11	**2-Methyl THF**	1.0	50.0
12	**2-Nitroethanol**	1.4	14.0
13	**Isovaleronitrile**	1.0	50.0
14	**2-Phenylethyl acetate**	1.0	50.0
15	***N*-Methylformanilide**	1.0	50.0
16	**Trimethylacetonitrile**	1.7	17.0
17	**Methyl anthranilate**	1.0	50.0
18	**Nitrobenzene**	1.0	50.0
19	**1-Methylimidazole**	1.0	50.0
20	**4-Formylmorpholine**	1.1	37.0
21	**Methanol**	1.0	100.0
22	**Ethanol**	1.0	100.0
23	***n*-propanol**	1.0	100.0
24	***iso*-propanol**	1.0	100.0
25	**Dichloromethane**	1.0	50.0
26	**Ethyl acetate**	1.0	25.0

**27**	**Adiponitrile**	1.0	25.0
**28**	***p*-Xylene**	1.0	25.0
**29**	**Dimethyl sulfoxide**	1.0	50.0
**30**	**Dimethyl formamide**	1.0	10.0
**31**	**Toluene**	1.0	25.0
**32**	**Acetic acid**	1.0	25.0

Table S2: Stock solutions of ROY used in ENaCt experiments.

S4. ENaCt Dispensing Protocols

Protocol 1a: Investigates all combinations of four crystallisation solvents, four encapsulating oils as well as no encapsulating oil per 96-well plate.

Protocol 1b: Investigates all combinations of four different solvent + antisolvent conditions, four encapsulating oils and no encapsulating oil per 96-well plate.

Protocol 2a: Investigates one solvent and oil combination per 96-well plate.

S4.1 Protocol 1a

Oil dispensing

1. Parent plate 1 is loaded with the prepared oils in the configuration shown (figure left) using a pasteur pippete.
2. Parent plate 1 is loaded onto the mosquito in position 1.
3. Oil dispense locations in crystallisation plate 1 are programmed into the SPT Latbtech software in the configuration shown (figure right).

Protocol 1a oil dispensing			
**Transfer type**	Aliquat	**Destination**	Position 5:<br>PDMSO, Fc-40: 2-6<br>Fomblin FY-1800, Mineral oil: 8-12
**Source**	Position 1		
**Dispense volume**	300 nL		
**Dispense type**	Contact	**Tip changing**	Between transfers

Table S3: Aspiration and dispensing parameters for oils used.

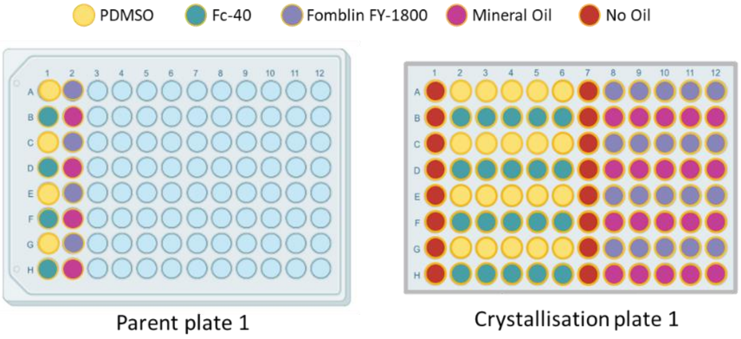

Figure S1: Aspiration and dispensing locations for oils.

Crystallisation solution dispensing

1. Parent plate 2 is loaded with the prepared crystallisation solutions in the configuration shown (figure left) using a micropipette.
2. Parent plate 1 is loaded onto the mosquito in position 3.
3. Crystallisation solution dispense locations in crystallisation plate 2 are programmed into the SPT Latbtech software in the configuration shown (figure right).

Protocol 1a crystallisation solution dispensing			
**Transfer type**	Aliquat	**Destination**	Position 5: 1-12
**Source**	Position 3	**Tip changing**	Always
**Dispense volume**	100 nL		
**Dispense type**	Contact		

Table S4: Aspiration and dispensing parameters for crystallisation solutions.

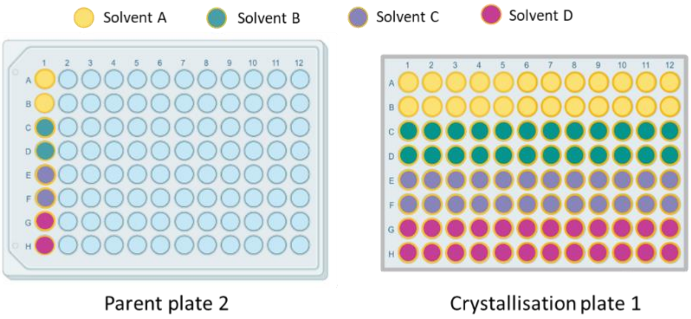

Figure S1: Aspiration and dispensing locations for crystallisation solutions.

S4.2. Protocol 1b

Oil dispensing

1. Parent plate 3 is loaded with the prepared oils in the configuration shown (figure left) using a pasteur pippete.
2. Parent plate 3 is loaded onto the mosquito in position 1.
3. Oil dispense locations in crystallisation plate 2 are programmed into the SPT Latbtech software in the configuration shown (figure right).

**Protocol 1b oil dispensing**			
**Transfer type**	Aliquat	**Destination**	Position 5:<br>PDMSO, Fc-40: 2-6<br>Fomblin FY-1800, Mineral oil: 8-12
**Source**	Position 1		
**Dispense volume**	300 nL		
**Dispense type**	Contact	**Tip changing**	Between transfers

Table S5: Aspiration and dispensing parameters for oils.

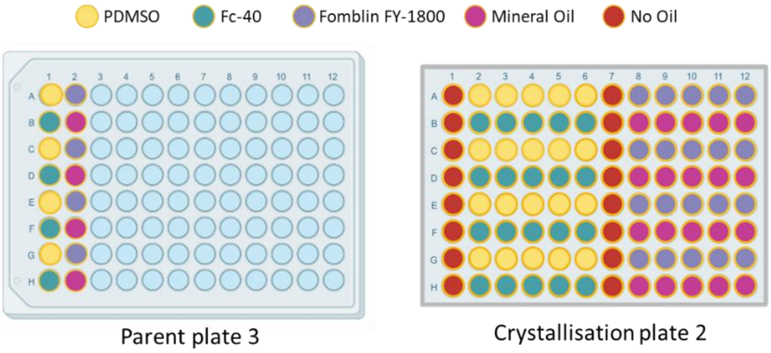

Figure S2: Aspiration and dispensing locations for oils used.

Crystallisation solution dispensing

1. Parent plate 4 is loaded with the prepared crystallisation solutions and antisolvent in the configuration shown (figure left) using a micropipette.
2. Parent plate 4 is loaded onto the mosquito in position 3.

3. Crystallisation solution and antisolvent dispense locations in crystallisation plate 2 are programmed into the SPT Latbtech software in the configuration shown (figure right).

Protocol 1b crystallisation solution dispensing			
**Transfer type**	Multi-aspirate	**Destination**	Position 5: 1-12
**Source**	Position 3	**Tip changing**	Always
**Dispense volume**	100 nL		
**Dispense type**	Contact		

Table S6: Aspiration and dispensing parameters for crystallisation solutions.

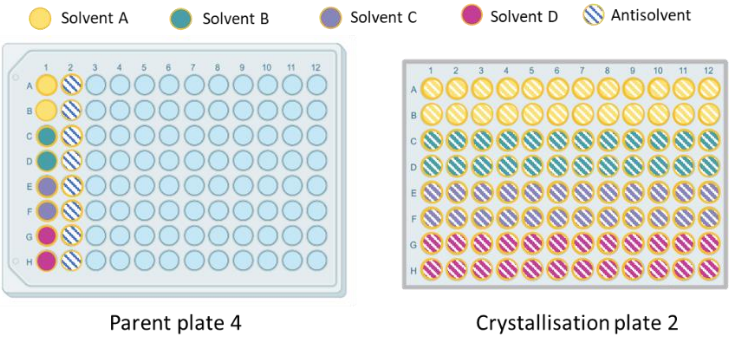

Figure S3: Aspiration and dispensing locations for crystallisation solutions.

S4.3. Protocol 2a

Oil dispensing

1. Parent plate 5 is loaded with a single oil in the configuration shown (figure left) using a pasteur pipette.
2. Parent plate 5 is loaded onto the mosquito in position 1.
3. Oil dispense locations in crystallisation plate 3 are programmed into the SPT Latbtech software in the configuration shown (figure right).

Protocol 2a oil dispensing			
**Transfer type**	Aliquat	**Destination**	Position 5:<br>Selected oil: 1-12
**Source**	Position 1		
**Dispense volume**	300 nL		
**Dispense type**	Contact	**Tip changing**	Between transfers

Table S7: Aspiration and dispensing parameters for oils.

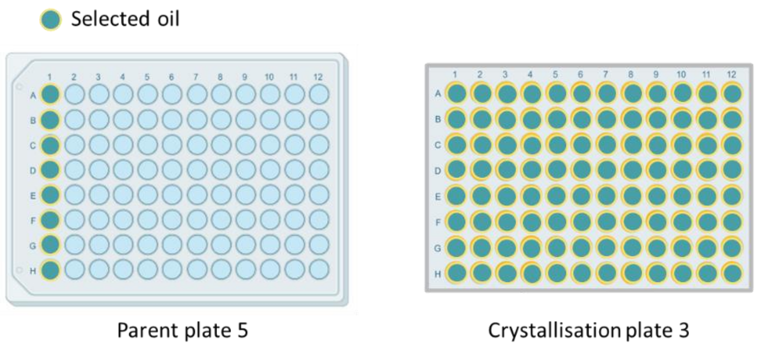

Figure S4: Aspiration and dispensing locations for oils.

Crystallisation solution dispensing

1. Parent plate 6 is loaded with the prepared crystallisation solutions and antisolvent in the configuration shown (figure left) using a micropipette.
2. Parent plate 6 is loaded onto the mosquito in position 3.
3. Crystallisation solution and antisolvent dispense locations in crystallisation plate 3 are programmed into the SPT Latbtech software in the configuration shown (figure right).

**Protocol 2a crystallisation solution dispensing**			
**Transfer type**	Aliquat	**Destination**	Position 5: 1-12
**Source**	Position 3	**Tip changing**	Always
**Dispense volume**	100 nL		
**Dispense type**	Contact		

Table S8: Aspiration and dispensing parameters for crystallisation solutions.

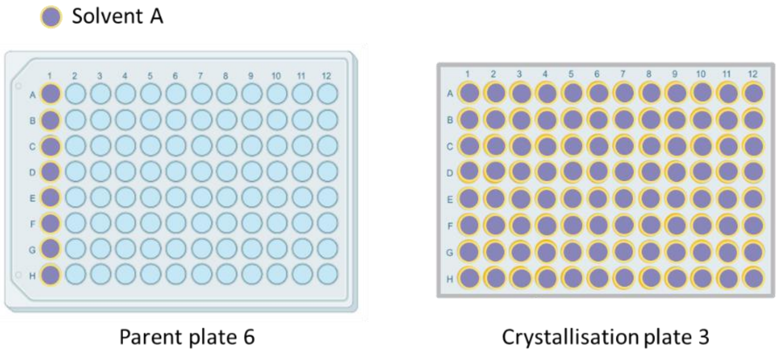

Figure S5: Aspiration and dispensing locations for crystallisation solutions.

S5. ENaCt Polymorph Screen

Using ROY stock solutions previously prepared, protocols 1a and 1b were used to prepared 16x 96 well plates, 8 containing solvent/oil experiments (P1A-P8A) and 8 containing solvent/antisolvent/oil experiments (P1B-P8B). After 7 days following experiment set-up, optical microscopy and unit cell determination by SC-XRD was used to identify the polymorphic form of identified crystals in each experimental well. Results are shown below in tables representing each 96-well plate, where each cell of the table contains which ROY forms were observed: Y, R, ON, ORP, YN, R18, O22, Y19, Solvate. Where cells are blank, no crystals suitable for polymorph identification and SC-XRD were observed. Where multiple ROY forms were observed in a single well, all forms are shown.

**P1A**	**1**	**2**	**3**	**4**	**5**	**6**	**7**	**8**	**9**	**10**	**11**	**12**
**Formamide**	Y	Y	Y	Y	Y	Y	Y	Y	Y	Y	Y	Y
**Formamide**	Y	Y	Y	Y	Y	Y	Y	Y	Y	Y	Y	Y
**1,4-Dioxane**	R	YN	ON						ON	ON	ON	
**1,4-Dioxane**		ON	ON	ON	ON			R	Y	ON	Y	Y,ON
**4-Methyl-2-pentanone**			YN	YN	YN			ON	ON	ON		
**4-Methyl-2-pentanone**						ON		YN		ON	ON	ON
**Benzonitrile**		ON	ON	ON	ON	YN						
**Benzonitrile**			YN	ON	ON	ON		ON	Y,ON	ON	ON	ON

**P2A**	**1**	**2**	**3**	**4**	**5**	**6**	**7**	**8**	**9**	**10**	**11**	**12**
**2,2,2-Trifluoro ethanol**							YN		Y04			
**2,2,2-Trifluoro ethanol**								Y	ON	Y04	Y04	ON
**2-Fluoro anisole**		ON,YN	ON,YN		Y	ON						
**2-Fluoro anisole**		R			ON	ON		R	YN	R	ON	ON
***n*-Butyl acetate**	Y	ON	Y04	ON	ON	ON					ON	
***n*-Butyl acetate**				ON				ON	ON	ON	R	R
**Dimethyl carbonate**		Y,ON	YN	YN	YN	Y,YN			YN	YN	YN,ON	ON
**Dimethyl carbonate**		ON		ON	ON	ON		Y	Y	YN	Y	ON

**P3A**	**1**	**2**	**3**	**4**	**5**	**6**	**7**	**8**	**9**	**10**	**11**	**12**
**Dichloroethane**			Y04	R	ON	ON						
**Dichloroethane**								ON	ON	ON	ON	ON
**1-Formyl pyrrolidine**		ON	ON	ON	ON	ON	ON					
**1-Formyl pyrrolidine**			R	R18	R18,ON		Y	ON	ON	ON	ON	ON
**2-Methyl THF**	Y,YN	Y,ON	Y04,ON		Y,ON							
**2-Methyl THF**			Y					ON	ON,YN	ON		ON
**2-Nitro ethanol**												
**2-Nitro ethanol**												Dimer

**P4A**	**1**	**2**	**3**	**4**	**5**	**6**	**7**	**8**	**9**	**10**	**11**	**12**
**Isovaleronitrile**		YN	YN	YN	YN	YN						
**Isovaleronitrile**		ON	ON	ON	ON	ON	R	ON	ON	ON	ON	
**2-Phenyl ethyl acetate**				Y			ON	ON	ON	ON	ON	
**2-Phenyl ethyl acetate**		R,ON	R18			R18		ON	ON	ON	ON	ON
***N*-methyl formanilide**	YN	YN	YN	YN,ON	YN,ON	YN						
***N*-methyl formanilide**	ON			ON	ON	ON	ON	ON	ON	ON	ON	ON
**Trimethyl acetonitrile**		YN	YN	YN	YN	YN						
**Trimethyl acetonitrile**										ON	ON	

**P5A**	**1**	**2**	**3**	**4**	**5**	**6**	**7**	**8**	**9**	**10**	**11**	**12**
**Methyl anthranilate**			Solvate						Solvate			
**Methyl anthranilate**	R			Solvate	Solvate	ON		Solvate	Solvate	Solvate	YN	YN
**Nitrobenzene**		ON	ON	ON	ON	ON	ON	ON	ON	ON	ON	Y
**Nitrobenzene**			ON	Y				ON	ON	ON	ON	ON
**1-Methyl imidazole**				Y								
**1-Methyl imidazole**		Y	Y	Y	ON	ON		ON		ON		ON
**4-Formyl morpholine**						YN						
**4-Formyl morpholine**			ON	ON	ON	ON			ON	Y	ON	ON

P6A	1	2	3	4	5	6	7	8	9	10	11	12
Methanol	Y	Y	Y	Y	Y	Y	Y	Y	Y	Y	Y	Y
Methanol	Y		Y	Y	Y	Y	Y	Y	Y	Y	Y,ON	Y
Ethanol	Y	Y	Y	Y	Y	Y	Y	Y	Y	Y		Y
Ethanol	Y	Y	Y	Y	Y	Y	Y	Y	Y	Y	Y	Y
propanol	Y	Y	Y	Y	Y	Y	Y	Y	Y	Y		Y
propanol	Y	Y	Y	Y	Y	Y	Y	Y	Y		Y	Y
Isopropanol		Y	Y	Y	Y	YN		Y	Y		Y	Y
Isopropanol		Y		Y				Y	Y	Y	Y	Y

P7A	1	2	3	4	5	6	7	8	9	10	11	12
Dichloromethane		Y	Y	Y,R	R,YN			R	R	R	R	R
Dichloromethane	R			ON			R	R	R	Y,R	R	Y
Ethyl acetate	Y,R	R,YN		ON			ON		Y		R	
Ethyl acetate	Y,ON	R,ON	R	ON	R	ON		R,ON	R,ON	Y,R,ON	R,ON	R,ON
Adiponitrile	ON		ON		ON	ON	ON		ON			
Adiponitrile	ON	ON	ON	ON	ON					ON		R18
p-Xylene	R		ON		ON	ON			Y	Y		
p-Xylene	Y	Y			Y	Y	Y	Y	Y	Y	Y	Y

P8A	1	2	3	4	5	6	7	8	9	10	11	12
DMSO		Y	R		R18,ON	ON	ON		Y	ON	R	ON
DMSO	ON	ON,R	ON	Y,ON	ON		ON	ON		ON	Y,R	O22
DMF		Y	ON	ON	Y	ON	Y	R	R	Y	ON	
DMF		Y	Y	Y	ON	ON			ON	Y	Y	ON
Toluene						ON					Y	YN
Toluene		ON	ON	ON	ON	ON		ON	ON	ON	ON	ON
Acetic acid	Y		Y	Y				Y		Y		
Acetic acid	Y	ON	Y					ON	ON	ON	ON	ON

P1B	1	2	3	4	5	6	7	8	9	10	11	12
Formamide	Y	Y	Y	Y	Y	Y	Y	Y	Y	Y	Y	Y
Formamide	Y	Y	Y	Y	Y	Y	Y	Y	Y	Y	Y	Y
1,4-Dioxane	R	YN				YN				YN		YN
1,4-Dioxane								YN	YN,ON	YN,ON	Y	YN
4-Methyl-2-pentanone		YN	YN		YN	YN		YN		YN	YN	YN
4-Methyl-2-pentanone								R,YN	R,YN	R,YN	R,YN	ON
Benzonitrile			YN		ON	YN	YN	YN	ON	ON	ON	ON
Benzonitrile			ON	ON				ON	Y,ON	ON	ON	ON

P2B	1	2	3	4	5	6	7	8	9	10	11	12
2,2,2-Trifluoro ethanol			Y04					ON		ON	ON	
2,2,2-Trifluoro ethanol										YN	ORP	
2-Fluoro anisole	Y	ON		R	ON,YN		YN					
2-Fluoro anisole		Y,ON	Y,ORP	ON,R	Y,ORP		YN	R,ON	ON	ON	ON	Y,ON
n-Butyl acetate		YN	YN	YN	YN	YN		ON				
n-Butyl acetate		YN	YN	YN	YN	YN		ON	R	ON	R,YN	ON
Dimethyl carbonate		ON	ON	ON	R	ON		ON				
Dimethyl carbonate		YN	YN			YN	R	ON	Y,R	ON	ON	ON

P3B	1	2	3	4	5	6	7	8	9	10	11	12
Dichloroethane	Y		YN	YN	YN	YN	ON			ON	ON	
Dichloroethane		R		Y				R	ON,R	R	ON	R
1-Formyl pyrrolidine	ON		ON				ON			YN		
1-Formyl pyrrolidine		ON,YN	Y	YN		Y04				Y	Y,Y04	Y,R
2-Methyl THF	ON	Y		Y	Y	ON,R		YN	ON			YN
2-Methyl THF			ON	Y	ON		ON	ON	ON		Y	
2-Nitro ethanol								YN				
2-Nitro ethanol		ON				Y,R						

P4B	1	2	3	4	5	6	7	8	9	10	11	12
Isovaleronitrile		Y	YN	YN	YN	Y,YN					ON	ON
Isovaleronitrile		ON	ON	Y	Y	ON		ON	R	Y	ON	ON
2-Phenyl ethyl acetate		ON		ON	ON	ON		ON	ON	ON	ON	ON
2-Phenyl ethyl acetate			Y					ON	R	ON	ON	ON
N-methyl formanilide		ON	ON	ON	ON	ON	ON					
N-methyl formanilide	YN	R,YN	YN	YN	YN		YN	ON	ON		ON	ON
Trimethyl acetonitrile	Y	YN	YN	YN	YN	YN						
Trimethyl acetonitrile		YN							Y	ON	ON	Y

P5B	1	2	3	4	5	6	7	8	9	10	11	12
Methyl anthranilate		ON	ON									
Methyl anthranilate			Y	Y	Y	Y				ON	ON	ON
Nitrobenzene		ON	Y	ON	Y			Y				
Nitrobenzene		ON		ON	ON	ON		ON	ON	Y	ON	ON
1-Methyl imidazole	Y	Y		Y								
1-Methyl imidazole		ON	ON	Y				Y	ON	ON	ON	ON
4-Formyl morpholine						YN			ON			
4-Formyl morpholine	ON	ON	ON		ON	Y,ON			Y	ON	ON	ON

P6B	1	2	3	4	5	6	7	8	9	10	11	12
Methanol	Y	Y	Y	Y	Y	Y	Y	Y	Y	Y	Y	Y
Methanol	Y	Y	Y	Y	Y	Y	Y	Y	Y	Y	Y	Y
Ethanol		Y	Y	Y	Y	Y	Y	Y	Y	Y	Y	Y
Ethanol	Y	Y	Y	Y	Y	Y	Y	Y	Y	Y	Y	Y
propanol	Y	Y	Y	Y	Y	Y	Y	Y	Y	Y	Y	Y
propanol	Y	Y	Y	Y	Y	Y	Y	Y	Y	Y	Y	Y
Isopropanol		Y	Y	Y	Y	Y	Y	Y	Y	Y	Y	Y
Isopropanol	Y	Y	Y	Y	Y	Y		Y	Y	Y	Y	Y

P7B	1	2	3	4	5	6	7	8	9	10	11	12
Dichloromethane		R	Y,R	Y		ON		R	R	Y,R	Y	Y
Dichloromethane		ON	YN,R		ON				R	Y	R	Y
Ethyl acetate		Y,R	Y	Y	Y			ON	Y		R,ON	
Ethyl acetate	Y,ON	R,ON	R	ON	R	ON		R,ON		R,ON	R,ON	R,ON
Adiponitrile		ON	ON		ON		ON		ON	ON		
Adiponitrile	ON			ON	ON			ON		ON	R	Y
p -Xylene			Y	ON	ON	ON			Y,R	R,ON		
p -Xylene		Y			Y	Y	Y	Y	Y	Y	Y	Y

P8B	1	2	3	4	5	6	7	8	9	10	11	12
DMSO		R18,ON	ON	R18,ON	R	R, ON		Y	R18,ON		R18,ON	R18,ON
DMSO	R18,ON		R18,ON	R,ON,R18	Y	R18,ON			Y	R18,ON	R18,ON	Y
DMF		Y19								ON		
DMF		ON		Y19				ON,Y19	ON,Y19	ON	ON,Y19	ON
Toluene	ON		ON	Y		ON		ON		ON	ON	Y
Toluene		ON	ON	ON	ON	ON			ON		Y,ON	ON
Acetic acid	ON						Y			ON	ON	
Acetic acid	ON	Y	ON		ON		ON	R	R,ON	R,ON	ON	ON

Figure S7: ROY polymorphs and other forms as identified within 96 well plates of initial ENaCt polymorph screen. White = forms identified optically, Yellow = forms identified by SCXRD unit cell determination.

S6. ROY Crystal Forms Observed by Crystallisation Condition

To identify conditions for selective access to specific ROY polymorphs and crystal forms, the number of wells containing each form was recorded for each crystallisation condition examined.

P1A	Formamide					1,4-Dioxane					4-Methyl-2-pentanone					Benzonitrile				
	No Oil	Pdmso	fc-40	FY	MO	No Oil	Pdmso	fc-40	FY	MO	No Oil	Pdmso	fc-40	FY	MO	No Oil	Pdmso	fc-40	FY	MO
Y	4	5	5	5	5	0	0	0	0	3	0	0	0	0	0	0	0	0	0	1
Y04	0	0	0	0	0	0	0	0	0	0	0	0	0	0	0	0	0	0	0	0
R	0	0	0	0	0	1	0	0	0	1	0	0	0	0	0	0	0	0	0	0
ON	0	0	0	0	0	0	1	4	3	2	0	0	1	3	3	0	4	3	0	5
YN	0	0	0	0	0	0	1	0	0	0	0	3	0	0	1	0	1	1	0	0
ORP	0	0	0	0	0	0	0	0	0	0	0	0	0	0	0	0	0	0	0	0
R18	0	0	0	0	0	0	0	0	0	0	0	0	0	0	0	0	0	0	0	0
Y19	0	0	0	0	0	0	0	0	0	0	0	0	0	0	0	0	0	0	0	0
OP	0	0	0	0	0	0	0	0	0	0	0	0	0	0	0	0	0	0	0	0
R05	0	0	0	0	0	0	0	0	0	0	0	0	0	0	0	0	0	0	0	0
RPL	0	0	0	0	0	0	0	0	0	0	0	0	0	0	0	0	0	0	0	0
PO13	0	0	0	0	0	0	0	0	0	0	0	0	0	0	0	0	0	0	0	0
YT04	0	0	0	0	0	0	0	0	0	0	0	0	0	0	0	0	0	0	0	0
O22	0	0	0	0	0	0	0	0	0	0	0	0	0	0	0	0	0	0	0	0
Dimer	0	0	0	0	0	0	0	0	0	0	0	0	0	0	0	0	0	0	0	0
Solvate	0	0	0	0	0	0	0	0	0	0	0	0	0	0	0	0	0	0	0	0
No crystal	0	0	0	0	0	3	3	1	2	0	4	2	4	2	0	4	0	1	5	0

P3A	Dichloroethanne					1 Formyl pyrrolidine					Tetrahydrothiophene 1-oxide					2 Nitro ethanol				
	No Oil	Pdmso	fc-40	FY	MO	No Oil	Pdmso	fc-40	FY	MO	No Oil	Pdmso	fc-40	FY	MO	No Oil	Pdmso	fc-40	FY	MO
Y	0	0	0	0	0	1	0	0	0	0	1	2	1	0	0	0	0	0	0	0
Y04	0	1	0	0	0	0	0	0	0	0	0	1	0	0	0	0	0	0	0	0
R	0	1	0	0	0	0	0	1	0	0	0	0	0	0	0	0	0	0	0	0
ON	0	2	0	0	5	1	5	1	0	5	0	3	0	0	4	0	0	0	0	0
YN	0	0	0	0	0	0	0	0	0	0	1	0	0	0	1	0	0	0	0	0
ORP	0	0	0	0	0	0	0	0	0	0	0	0	0	0	0	0	0	0	0	0
R18	0	0	0	0	0	0	0	2	0	0	0	0	0	0	0	0	0	0	0	0
Y19	0	0	0	0	0	0	0	0	0	0	0	0	0	0	0	0	0	0	0	0
OP	0	0	0	0	0	0	0	0	0	0	0	0	0	0	0	0	0	0	0	0
R05	0	0	0	0	0	0	0	0	0	0	0	0	0	0	0	0	0	0	0	0
RPL	0	0	0	0	0	0	0	0	0	0	0	0	0	0	0	0	0	0	0	0
PO13	0	0	0	0	0	0	0	0	0	0	0	0	0	0	0	0	0	0	0	0
YT04	0	0	0	0	0	0	0	0	0	0	0	0	0	0	0	0	0	0	0	0
O22	0	0	0	0	0	0	0	0	0	0	0	0	0	0	0	0	0	0	0	0
Dimer	0	0	0	0	0	0	0	0	0	0	0	0	0	0	0	0	0	0	0	1
Solvate	0	0	0	0	0	0	0	0	0	0	0	0	0	0	0	0	0	0	0	0
No crystal	4	1	5	5	0	2	0	2	5	0	3	2	4	5	1	4	5	5	5	4

P4A	Isovaleronitrile					2 Phenyl ethyl acetate					*N*-methyl formanilide					Trimethyl acetonitrile				
	No Oil	Pdmso	fc-40	FY	MO	No Oil	Pdmso	fc-40	FY	MO	No Oil	Pdmso	fc-40	FY	MO	No Oil	Pdmso	fc-40	FY	MO
Y	0	0	0	0	0	0	1	0	0	0	0	0	0	0	0	0	0	0	0	0
Y04	0	0	0	0	0	0	0	0	0	0	0	0	0	0	0	0	0	0	0	0
R	1	0	0	0	0	0	0	1	0	0	0	0	0	0	0	0	0	0	0	0
ON	0	0	5	0	4	1	0	1	4	5	2	2	3	0	5	0	0	0	0	2
YN	0	5	0	0	0	0	0	0	0	0	1	5	0	0	0	0	5	0	0	0
ORP	0	0	0	0	0	0	0	0	0	0	0	0	0	0	0	0	0	0	0	0
R18	0	0	0	0	0	0	0	2	0	0	0	0	0	0	0	0	0	0	0	0
Y19	0	0	0	0	0	0	0	0	0	0	0	0	0	0	0	0	0	0	0	0
OP	0	0	0	0	0	0	0	0	0	0	0	0	0	0	0	0	0	0	0	0
R05	0	0	0	0	0	0	0	0	0	0	0	0	0	0	0	0	0	0	0	0
RPL	0	0	0	0	0	0	0	0	0	0	0	0	0	0	0	0	0	0	0	0
PO13	0	0	0	0	0	0	0	0	0	0	0	0	0	0	0	0	0	0	0	0
YT04	0	0	0	0	0	0	0	0	0	0	0	0	0	0	0	0	0	0	0	0
O22	0	0	0	0	0	0	0	0	0	0	0	0	0	0	0	0	0	0	0	0
Dimer	0	0	0	0	0	0	0	0	0	0	0	0	0	0	0	0	0	0	0	0
Solvate	0	0	0	0	0	0	0	0	0	0	0	0	0	0	0	0	0	0	0	0
No crystal	3	0	0	5	1	3	4	0	1	0	1	0	2	5	0	4	0	5	5	3

P5A	Methyl anthranilate					Nitrobenzene					1 methyl imidazole					4 formyl morpholine				
	No Oil	Pdmso	fc-40	FY	MO	No Oil	Pdmso	fc-40	FY	MO	No Oil	Pdmso	fc-40	FY	MO	No Oil	Pdmso	fc-40	FY	MO
Y	0	0	0	0	0	0	0	1	1	0	0	1	3	0	0	0	0	0	0	1
Y04	0	0	0	0	0	0	0	0	0	0	0	0	0	0	0	0	0	0	0	0
R	1	0	0	0	0	0	0	0	0	0	0	0	0	0	0	0	0	0	0	0
ON	0	0	1	0	0	1	5	1	4	5	0	0	2	0	3	0	0	4	0	3
YN	0	0	0	0	2	0	0	0	0	0	0	0	0	0	0	0	1	0	0	0
ORP	0	0	0	0	0	0	0	0	0	0	0	0	0	0	0	0	0	0	0	0
R18	0	0	0	0	0	0	0	0	0	0	0	0	0	0	0	0	0	0	0	0
Y19	0	0	0	0	0	0	0	0	0	0	0	0	0	0	0	0	0	0	0	0
OP	0	0	0	0	0	0	0	0	0	0	0	0	0	0	0	0	0	0	0	0
R05	0	0	0	0	0	0	0	0	0	0	0	0	0	0	0	0	0	0	0	0
RPL	0	0	0	0	0	0	0	0	0	0	0	0	0	0	0	0	0	0	0	0
PO13	0	0	0	0	0	0	0	0	0	0	0	0	0	0	0	0	0	0	0	0
YT04	0	0	0	0	0	0	0	0	0	0	0	0	0	0	0	0	0	0	0	0
O22	0	0	0	0	0	0	0	0	0	0	0	0	0	0	0	0	0	0	0	0
Dimer	0	0	0	0	0	0	0	0	0	0	0	0	0	0	0	0	0	0	0	0
Solvate	0	1	2	1	3	0	0	0	0	0	0	0	0	0	0	0	0	0	0	0
No crystal	3	4	2	4	0	3	0	3	0	0	4	4	0	5	3	4	4	1	5	1

P6A	Methanol					Ethanol					n-Propanol					Isopropanol				
	No Oil	Pdmso	fc-40	FY	MO	No Oil	Pdmso	fc-40	FY	MO	No Oil	Pdmso	fc-40	FY	MO	No Oil	Pdmso	fc-40	FY	MO
Y	4	5	4	5	5	4	5	5	4	5	4	5	5	4	4	0	4	2	4	5
Y04	0	0	0	0	0	0	0	0	0	0	0	0	0	0	0	0	0	0	0	0
R	0	0	0	0	0	0	0	0	0	0	0	0	0	0	0	0	0	0	0	0
ON	0	0	0	0	1	0	0	0	0	0	0	0	0	0	0	0	0	0	0	0
YN	0	0	0	0	0	0	0	0	0	0	0	0	0	0	0	0	1	0	0	0
ORP	0	0	0	0	0	0	0	0	0	0	0	0	0	0	0	0	0	0	0	0
R18	0	0	0	0	0	0	0	0	0	0	0	0	0	0	0	0	0	0	0	0
Y19	0	0	0	0	0	0	0	0	0	0	0	0	0	0	0	0	0	0	0	0
OP	0	0	0	0	0	0	0	0	0	0	0	0	0	0	0	0	0	0	0	0
R05	0	0	0	0	0	0	0	0	0	0	0	0	0	0	0	0	0	0	0	0
RPL	0	0	0	0	0	0	0	0	0	0	0	0	0	0	0	0	0	0	0	0
PO13	0	0	0	0	0	0	0	0	0	0	0	0	0	0	0	0	0	0	0	0
YT04	0	0	0	0	0	0	0	0	0	0	0	0	0	0	0	0	0	0	0	0
O22	0	0	0	0	0	0	0	0	0	0	0	0	0	0	0	0	0	0	0	0
Dimer	0	0	0	0	0	0	0	0	0	0	0	0	0	0	0	0	0	0	0	0
Solvate	0	0	0	0	0	0	0	0	0	0	0	0	0	0	0	0	0	0	0	0
No crystal	0	0	1	0	0	0	0	0	1	0	0	0	0	1	1	4	0	3	1	0

P7A	Dichloromethane					Ethyl acetate					Adiponitrile					p-Xylene				
	No Oil	Pdmso	fc-40	FY	MO	No Oil	Pdmso	fc-40	FY	MO	No Oil	Pdmso	fc-40	FY	MO	No Oil	Pdmso	fc-40	FY	MO
Y	0	3	0	0	2	2	0	0	1	1	0	0	0	0	0	2	0	3	2	5
Y04	0	0	0	0	0	0	0	0	0	0	0	0	0	0	0	0	0	0	0	0
R	2	2	0	5	4	1	1	3	1	5	0	0	0	0	0	1	0	0	0	0
ON	0	0	1	0	0	2	1	3	0	5	3	3	4	1	1	0	3	0	0	0
YN	0	1	0	0	0	0	1	0	0	0	0	0	0	0	0	0	0	0	0	0
ORP	0	0	0	0	0	0	0	0	0	0	0	0	0	0	0	0	0	0	0	0
R18	0	0	0	0	0	0	0	0	0	0	0	0	0	0	1	0	0	0	0	0
Y19	0	0	0	0	0	0	0	0	0	0	0	0	0	0	0	0	0	0	0	0
OP	0	0	0	0	0	0	0	0	0	0	0	0	0	0	0	0	0	0	0	0
R05	0	0	0	0	0	0	0	0	0	0	0	0	0	0	0	0	0	0	0	0
RPL	0	0	0	0	0	0	0	0	0	0	0	0	0	0	0	0	0	0	0	0
PO13	0	0	0	0	0	0	0	0	0	0	0	0	0	0	0	0	0	0	0	0
YT04	0	0	0	0	0	0	0	0	0	0	0	0	0	0	0	0	0	0	0	0
O22	0	0	0	0	0	0	0	0	0	0	0	0	0	0	0	0	0	0	0	0
Dimer	0	0	0	0	0	0	0	0	0	0	0	0	0	0	0	0	0	0	0	0
Solvate	0	0	0	0	0	0	0	0	0	0	0	0	0	0	0	0	0	0	0	0
No crystal	2	1	4	0	0	1	3	0	3	0	1	2	1	4	3	1	2	2	3	0

P8A	Dimethyl sulfoxide					Dimethyl formamide					Toluene					Acetic acid				
	No Oil	Pdmso	fc-40	FY	MO	No Oil	Pdmso	fc-40	FY	MO	No Oil	Pdmso	fc-40	FY	MO	No Oil	Pdmso	fc-40	FY	MO
Y	0	1	1	1	1	1	2	3	1	2	0	0	0	1	0	2	2	1	2	0
Y04	0	0	0	0	0	0	0	0	0	0	0	0	0	0	0	0	0	0	0	0
R	0	1	1	1	1	0	0	0	2	0	0	0	0	0	0	0	0	0	0	0
ON	3	2	4	2	2	0	3	2	1	2	0	1	5	0	5	0	0	1	0	5
YN	0	0	0	0	0	0	0	0	0	0	0	0	0	1	0	0	0	0	0	0
ORP	0	0	0	0	0	0	0	0	0	0	0	0	0	0	0	0	0	0	0	0
R18	0	1	0	0	0	0	0	0	0	0	0	0	0	0	0	0	0	0	0	0
Y19	0	0	0	0	0	0	0	0	0	0	0	0	0	0	0	0	0	0	0	0
OP	0	0	0	0	0	0	0	0	0	0	0	0	0	0	0	0	0	0	0	0
R05	0	0	0	0	0	0	0	0	0	0	0	0	0	0	0	0	0	0	0	0
RPL	0	0	0	0	0	0	0	0	0	0	0	0	0	0	0	0	0	0	0	0
PO13	0	0	0	0	0	0	0	0	0	0	0	0	0	0	0	0	0	0	0	0
YT04	0	0	0	0	0	0	0	0	0	0	0	0	0	0	0	0	0	0	0	0
O22	0	0	0	0	1	0	0	0	0	0	0	0	0	0	0	0	0	0	0	0
Dimer	0	0	0	0	0	0	0	0	0	0	0	0	0	0	0	0	0	0	0	0
Solvate	0	0	0	0	0	0	0	0	0	0	0	0	0	0	0	0	0	0	0	0
No crystal	1	1	1	1	1	3	0	0	1	1	4	4	0	3	0	2	3	3	3	0

P1B	Formamide/water					1,4-Dioxane/water					4-Methyl-2-pentanone/water					Benzonitrile/water				
	No Oil	Pdmso	fc-40	FY	MO	No Oil	Pdmso	fc-40	FY	MO	No Oil	Pdmso	fc-40	FY	MO	No Oil	Pdmso	fc-40	FY	MO
Y	4	5	5	5	5	0	0	0	0	1	0	0	0	0	0	0	0	0	0	1
Y04	0	0	0	0	0	0	0	0	0	0	0	0	0	0	0	0	0	0	0	0
R	0	0	0	0	0	1	0	0	0	0	0	0	0	0	4	0	0	0	0	0
ON	0	0	0	0	0	0	0	0	0	2	0	0	0	0	1	0	1	2	4	0
YN	0	0	0	0	0	0	2	0	2	4	0	4	0	4	4	1	2	0	1	5
ORP	0	0	0	0	0	0	0	0	0	0	0	0	0	0	0	0	0	0	0	0
R18	0	0	0	0	0	0	0	0	0	0	0	0	0	0	0	0	0	0	0	0
Y19	0	0	0	0	0	0	0	0	0	0	0	0	0	0	0	0	0	0	0	0
OP	0	0	0	0	0	0	0	0	0	0	0	0	0	0	0	0	0	0	0	0
R05	0	0	0	0	0	0	0	0	0	0	0	0	0	0	0	0	0	0	0	0
RPL	0	0	0	0	0	0	0	0	0	0	0	0	0	0	0	0	0	0	0	0
PO13	0	0	0	0	0	0	0	0	0	0	0	0	0	0	0	0	0	0	0	0
YT04	0	0	0	0	0	0	0	0	0	0	0	0	0	0	0	0	0	0	0	0
O22	0	0	0	0	0	0	0	0	0	0	0	0	0	0	0	0	0	0	0	0
Dimer	0	0	0	0	0	0	0	0	0	0	0	0	0	0	0	0	0	0	0	0
Solvate	0	0	0	0	0	0	0	0	0	0	0	0	0	0	0	0	0	0	0	0
No crystal	0	0	0	0	0	3	3	5	0	0	4	1	5	5	0	3	0	3	0	0

P2B	2,2,2-Trifluoroethanol/water					2-Fluoroanisole/water					*n*-Butyl acetate/water					Dimethyl carbonate/water				
	No Oil	Pdmso	fc-40	FY	MO	No Oil	Pdmso	fc-40	FY	MO	No Oil	Pdmso	fc-40	FY	MO	No Oil	Pdmso	fc-40	FY	MO
Y	0	0	0	0	0	1	0	3	0	1	0	0	0	0	0	0	0	0	0	1
Y04	0	1	0	0	0	0	0	0	0	0	0	0	0	0	0	0	0	0	0	0
R	0	0	0	0	0	0	1	1	0	1	0	0	0	0	2	1	1	0	0	1
ON	0	0	0	3	0	0	2	2	0	5	0	0	0	1	3	0	4	0	0	4
YN	0	0	0	0	1	2	1	0	0	0	0	5	5	0	1	0	0	3	0	0
ORP	0	0	0	0	1	0	0	2	0	0	0	0	0	0	0	0	0	0	0	0
R18	0	0	0	0	0	0	0	0	0	0	0	0	0	0	0	0	0	0	0	0
Y19	0	0	0	0	0	0	0	0	0	0	0	0	0	0	0	0	0	0	0	0
OP	0	0	0	0	0	0	0	0	0	0	0	0	0	0	0	0	0	0	0	0
R05	0	0	0	0	0	0	0	0	0	0	0	0	0	0	0	0	0	0	0	0
RPL	0	0	0	0	0	0	0	0	0	0	0	0	0	0	0	0	0	0	0	0
PO13	0	0	0	0	0	0	0	0	0	0	0	0	0	0	0	0	0	0	0	0
YT04	0	0	0	0	0	0	0	0	0	0	0	0	0	0	0	0	0	0	0	0
O22	0	0	0	0	0	0	0	0	0	0	0	0	0	0	0	0	0	0	0	0
Dimer	0	0	0	0	0	0	0	0	0	0	0	0	0	0	0	0	0	0	0	0
Solvate	0	0	0	0	0	0	0	0	0	0	0	0	0	0	0	0	0	0	0	0
No crystal	4	4	5	2	3	1	0	1	5	0	4	0	0	4	0	3	0	2	5	0

P3B	Dichloroethane/water					1-Formyl pyrrolidine/water					2-Methyl THF/water					2-Nitro ethanol/water				
	No Oil	Pdmso	fc-40	FY	MO	No Oil	Pdmso	fc-40	FY	MO	No Oil	Pdmso	fc-40	FY	MO	No Oil	Pdmso	fc-40	FY	MO
Y	1	0	1	0	0	0	0	1	0	3	0	3	1	0	1	0	0	1	0	0
Y04	0	0	0	0	0	0	0	1	0	1	0	0	0	0	0	0	0	0	0	0
R	0	0	1	0	4	0	0	0	0	1	0	0	0	0	0	0	0	1	0	0
ON	1	0	0	2	1	2	1	1	0	0	2	1	2	1	2	0	0	1	1	0
YN	0	4	0	0	0	0	0	2	1	0	0	0	0	2	0	0	0	0	0	0
ORP	0	0	0	0	0	0	0	0	0	0	0	0	0	0	0	0	0	0	0	0
R18	0	0	0	0	0	0	0	0	0	0	0	0	0	0	0	0	0	0	0	0
Y19	0	0	0	0	0	0	0	0	0	0	0	0	0	0	0	0	0	0	0	0
OP	0	0	0	0	0	0	0	0	0	0	0	0	0	0	0	0	0	0	0	0
R05	0	0	0	0	0	0	0	0	0	0	0	0	0	0	0	0	0	0	0	0
RPL	0	0	0	0	0	0	0	0	0	0	0	0	0	0	0	0	0	0	0	0
PO13	0	0	0	0	0	0	0	0	0	0	0	0	0	0	0	0	0	0	0	0
YT04	0	0	0	0	0	0	0	0	0	0	0	0	0	0	0	0	0	0	0	0
O22	0	0	0	0	0	0	0	0	0	0	0	0	0	0	0	0	0	0	0	0
Dimer	0	0	0	0	0	0	0	0	0	0	0	0	0	0	0	0	0	0	0	0
Solvate	0	0	0	0	0	0	0	0	0	0	0	0	0	0	0	0	0	0	0	0
No crystal	2	1	3	3	0	2	4	1	4	2	3	1	2	2	1	4	5	3	4	5

P4B	Isovaleronitrile/water					2-Phenyl ethyl acetate/water					*n*-Methyl formanilide/water					Trimethyl acetonitrile/water				
	No Oil	Pdmso	fc-40	FY	MO	No Oil	Pdmso	fc-40	FY	MO	No Oil	Pdmso	fc-40	FY	MO	No Oil	Pdmso	fc-40	FY	MO
Y	0	2	2	0	1	0	0	1	0	0	0	0	0	0	0	1	0	0	0	2
Y04	0	0	0	0	0	0	0	0	0	0	0	0	0	0	0	0	0	0	0	0
R	0	0	0	0	1	0	0	0	0	1	0	0	1	0	0	0	0	0	0	0
ON	0	0	3	2	3	0	4	0	5	4	1	5	0	0	4	0	0	0	0	2
YN	0	4	0	0	0	0	0	0	0	0	2	0	4	0	0	0	5	1	0	0
ORP	0	0	0	0	0	0	0	0	0	0	0	0	0	0	0	0	0	0	0	0
R18	0	0	0	0	0	0	0	0	0	0	0	0	0	0	0	0	0	0	0	0
Y19	0	0	0	0	0	0	0	0	0	0	0	0	0	0	0	0	0	0	0	0
OP	0	0	0	0	0	0	0	0	0	0	0	0	0	0	0	0	0	0	0	0
R05	0	0	0	0	0	0	0	0	0	0	0	0	0	0	0	0	0	0	0	0
RPL	0	0	0	0	0	0	0	0	0	0	0	0	0	0	0	0	0	0	0	0
PO13	0	0	0	0	0	0	0	0	0	0	0	0	0	0	0	0	0	0	0	0
YT04	0	0	0	0	0	0	0	0	0	0	0	0	0	0	0	0	0	0	0	0
O22	0	0	0	0	0	0	0	0	0	0	0	0	0	0	0	0	0	0	0	0
Dimer	0	0	0	0	0	0	0	0	0	0	0	0	0	0	0	0	0	0	0	0
Solvate	0	0	0	0	0	0	0	0	0	0	0	0	0	0	0	0	0	0	0	0
No crystal	4	0	0	3	0	4	1	4	1	0	1	0	2	5	1	3	0	0	5	1

P5B	Methyl anthranilate/water					Nitrobenzene/water					1-Methyl imidazole/water					4-Methyl morpholine/water				
	No Oil	Pdmso	fc-40	FY	MO	No Oil	Pdmso	fc-40	FY	MO	No Oil	Pdmso	fc-40	FY	MO	No Oil	Pdmso	fc-40	FY	MO
Y	0	0	4	0	0	0	2	0	1	1	1	2	1	0	1	0	0	1	0	1
Y04	0	0	0	0	0	0	0	0	0	0	0	0	0	0	0	0	0	0	0	0
R	0	0	0	0	0	0	0	0	0	0	0	0	0	0	0	0	0	0	0	0
ON	0	2	0	0	3	0	2	4	0	4	0	0	2	0	4	1	0	4	1	3
YN	0	0	0	0	0	0	0	0	0	0	0	0	0	0	0	0	1	0	0	0
ORP	0	0	0	0	0	0	0	0	0	0	0	0	0	0	0	0	0	0	0	0
R18	0	0	0	0	0	0	0	0	0	0	0	0	0	0	0	0	0	0	0	0
Y19	0	0	0	0	0	0	0	0	0	0	0	0	0	0	0	0	0	0	0	0
OP	0	0	0	0	0	0	0	0	0	0	0	0	0	0	0	0	0	0	0	0
R05	0	0	0	0	0	0	0	0	0	0	0	0	0	0	0	0	0	0	0	0
RPL	0	0	0	0	0	0	0	0	0	0	0	0	0	0	0	0	0	0	0	0
PO13	0	0	0	0	0	0	0	0	0	0	0	0	0	0	0	0	0	0	0	0
YT04	0	0	0	0	0	0	0	0	0	0	0	0	0	0	0	0	0	0	0	0
O22	0	0	0	0	0	0	0	0	0	0	0	0	0	0	0	0	0	0	0	0
Dimer	0	0	0	0	0	0	0	0	0	0	0	0	0	0	0	0	0	0	0	0
Solvate	0	0	0	0	0	0	0	0	0	0	0	0	0	0	0	0	0	0	0	0
No crystal	4	3	1	5	2	4	1	1	4	0	3	3	2	5	0	3	4	0	4	1

P6B	Methanol/water					Ethanol/water					*n*-Propanol/water					*i*-Propanol/water				
	**No Oil**	**Pdmso**	**fc-40**	**FY**	**MO**	**No Oil**	**Pdmso**	**fc-40**	**FY**	**MO**	**No Oil**	**Pdmso**	**fc-40**	**FY**	**MO**	**No Oil**	**Pdmso**	**fc-40**	**FY**	**MO**
**Y**	4	5	5	5	5	3	5	5	5	5	4	5	5	5	5	2	5	5	5	5
**Y04**	0	0	0	0	0	0	0	0	0	0	0	0	0	0	0	0	0	0	0	0
**R**	0	0	0	0	0	0	0	0	0	0	0	0	0	0	0	0	0	0	0	0
**ON**	0	0	0	0	0	0	0	0	0	0	0	0	0	0	0	0	0	0	0	0
**YN**	0	0	0	0	0	0	0	0	0	0	0	0	0	0	0	0	0	0	0	0
**ORP**	0	0	0	0	0	0	0	0	0	0	0	0	0	0	0	0	0	0	0	0
**R18**	0	0	0	0	0	0	0	0	0	0	0	0	0	0	0	0	0	0	0	0
**Y19**	0	0	0	0	0	0	0	0	0	0	0	0	0	0	0	0	0	0	0	0
**OP**	0	0	0	0	0	0	0	0	0	0	0	0	0	0	0	0	0	0	0	0
**R05**	0	0	0	0	0	0	0	0	0	0	0	0	0	0	0	0	0	0	0	0
**RPL**	0	0	0	0	0	0	0	0	0	0	0	0	0	0	0	0	0	0	0	0
**PO13**	0	0	0	0	0	0	0	0	0	0	0	0	0	0	0	0	0	0	0	0
**YT04**	0	0	0	0	0	0	0	0	0	0	0	0	0	0	0	0	0	0	0	0
**O22**	0	0	0	0	0	0	0	0	0	0	0	0	0	0	0	0	0	0	0	0
**Dimer**	0	0	0	0	0	0	0	0	0	0	0	0	0	0	0	0	0	0	0	0
**Solvate**	0	0	0	0	0	0	0	0	0	0	0	0	0	0	0	0	0	0	0	0
**No crystal**	0	0	0	0	0	1	0	0	0	0	0	0	0	0	0	2	0	0	0	0

P7B	Dichloromethane/water					Ethyl acetate/water					Adiponitrile/water					*p*-Xylene/water				
	No Oil	Pdmso	fc-40	FY	MO	No Oil	Pdmso	fc-40	FY	MO	No Oil	Pdmso	fc-40	FY	MO	No Oil	Pdmso	fc-40	FY	MO
**Y**	0	2	0	3	2	1	4	0	1	0	0	0	0	0	1	1	1	3	1	5
**Y04**	0	0	0	0	0	0	0	0	0	0	0	0	0	0	0	0	0	0	0	0
**R**	0	2	1	3	2	0	1	3	1	3	0	0	0	0	1	0	0	0	2	0
**ON**	0	1	2	0	0	1	0	3	2	3	2	3	2	2	2	0	3	0	1	0
**YN**	0	0	1	0	0	0	0	0	0	0	0	0	0	0	0	0	0	0	0	0
**ORP**	0	0	0	0	0	0	0	0	0	0	0	0	0	0	0	0	0	0	0	0
**R18**	0	0	0	0	0	0	0	0	0	0	0	0	0	0	0	0	0	0	0	0
**Y19**	0	0	0	0	0	0	0	0	0	0	0	0	0	0	0	0	0	0	0	0
**OP**	0	0	0	0	0	0	0	0	0	0	0	0	0	0	0	0	0	0	0	0
**R05**	0	0	0	0	0	0	0	0	0	0	0	0	0	0	0	0	0	0	0	0
**RPL**	0	0	0	0	0	0	0	0	0	0	0	0	0	0	0	0	0	0	0	0
**PO13**	0	0	0	0	0	0	0	0	0	0	0	0	0	0	0	0	0	0	0	0
**YT04**	0	0	0	0	0	0	0	0	0	0	0	0	0	0	0	0	0	0	0	0
**O22**	0	0	0	0	0	0	0	0	0	0	0	0	0	0	0	0	0	0	0	0
**Dimer**	0	0	0	0	0	0	0	0	0	0	0	0	0	0	0	0	0	0	0	0
**Solvate**	0	0	0	0	0	0	0	0	0	0	0	0	0	0	0	0	0	0	0	0
**No crystal**	4	0	2	0	1	3	1	0	1	1	2	2	3	3	1	3	1	2	2	0

P8B	DMSO/water					DMF/water					Toluene/water					Acetic acid/water				
	No Oil	Pdmso	fc-40	FY	MO	No Oil	Pdmso	fc-40	FY	MO	No Oil	Pdmso	fc-40	FY	MO	No Oil	Pdmso	fc-40	FY	MO
**Y**	0	0	1	1	2	0	0	0	0	0	0	1	0	1	1	1	0	1	0	0
**Y04**	0	0	0	0	0	0	0	0	0	0	0	0	0	0	0	0	0	0	0	0
**R**	0	2	1	0	0	0	0	0	0	0	0	0	0	0	0	0	0	0	0	3
**ON**	1	4	3	3	2	0	0	1	1	5	1	2	5	3	3	3	0	2	2	4
**YN**	0	0	0	0	0	0	0	0	0	0	0	0	0	0	0	0	0	0	0	0
**ORP**	0	0	0	0	0	0	0	0	0	0	0	0	0	0	0	0	0	0	0	0
**R18**	1	2	3	3	2	0	0	0	0	0	0	0	0	0	0	0	0	0	0	0
**Y19**	0	0	0	0	0	0	1	1	0	3	0	0	0	0	0	0	0	0	0	0
**OP**	0	0	0	0	0	0	0	0	0	0	0	0	0	0	0	0	0	0	0	0
**R05**	0	0	0	0	0	0	0	0	0	0	0	0	0	0	0	0	0	0	0	0
**RPL**	0	0	0	0	0	0	0	0	0	0	0	0	0	0	0	0	0	0	0	0
**PO13**	0	0	0	0	0	0	0	0	0	0	0	0	0	0	0	0	0	0	0	0
**YT04**	0	0	0	0	0	0	0	0	0	0	0	0	0	0	0	0	0	0	0	0
**O22**	0	0	0	0	0	0	0	0	0	0	0	0	0	0	0	0	0	0	0	0
**Dimer**	0	0	0	0	0	0	0	0	0	0	0	0	0	0	0	0	0	0	0	0
**Solvate**	0	0	0	0	0	0	0	0	0	0	0	0	0	0	0	0	0	0	0	0
**No crystal**	3	0	1	1	1	4	2	3	4	0	3	2	0	1	1	2	5	2	3	0

Figure S9: Number of each ROY *crystal form* observed for each of the 320 crystallisation conditions investigated. *Shades of green used to show conditions where an increasing number of the 5 experimental replicates contain the same form.*

S7. Improved Crystallisation of ROY·methyl anthranilate solvate by Seeded ENaCt

Seed crystals of the ROY·methyl anthranilate solvate were harvested from wells of the targeted follow-up plate. Crystals were ground between two glass microscope slides to produce a microcrystalline seed stock. Seed stock was then suspended within the mineral oil used to encapsulate the crystallisation solutions. This seeded oil was then employed in protocol 2a, using 50 mg/mL ROY in methyl anthranilate, for set up one 96 well plate containing a single experimental condition. Crystallisation of single crystals suitable for single crystal X-ray diffraction were observed after 1 day.

solvate plate seeded	1	2	3	4	5	6	7	8	9	10	11	12
A	Solvate	Solvate	Solvate	Solvate	Solvate	Solvate	Solvate	Solvate	Solvate	Solvate	Solvate	Solvate
B	Solvate	Solvate	Solvate	Solvate	Solvate	Solvate	Solvate	Solvate	Solvate	Solvate	Solvate	Solvate
C	Solvate	Solvate	Solvate	Solvate	Solvate	Solvate	Solvate	Solvate	Solvate	Solvate	Solvate	Solvate
D	Solvate	Solvate	Solvate	Solvate	Solvate	Solvate	Solvate	Solvate	Solvate	Solvate	Solvate	Solvate
E	Solvate	Solvate	Solvate	Solvate	Solvate	Solvate	Solvate	Solvate	Solvate	Solvate	Solvate	Solvate
F	Solvate	Solvate	Solvate	Solvate	Solvate	Solvate	Solvate	Solvate	Solvate	Solvate	Solvate	Solvate
G	Solvate	Solvate	Solvate	Solvate	Solvate	Solvate	Solvate	Solvate	Solvate	Solvate	Solvate	Solvate
H	Solvate	Solvate	Solvate	Solvate	Solvate	Solvate	Solvate	Solvate	Solvate	Solvate	Solvate	Solvate

Figure S8: ROY solvate as identified within 96 well plates of targeted screen. White = forms identified optically, Yellow = forms identified by SCXRD unit cell determination.

S8. Comparison of O22 with the CSP Structure Rank #24

S8.1 Comparison by Mercury 4.0[S7]

Using the Mercury 4.0 Crystal Packing Similarity, a 15-molecule cluster for both O22 and CSP Structure Rank #24 was calculated using the default tolerance values (20 % for distances between overlayed molecules and 20 degrees for angles between overlayed molecules).[S9] Successful overlay of all 15 molecules was obtained with a root-mean-square deviation (RMSD) of 0.137 Å.

S8.2 Comparison by Crystal CMP[S8]

Using Crystal CMP, a comparison of a 15-molecule cluster of both O22 and CSP Structure Rank #24 was undertaken, using the smiles string (N(C1SCCC1C)C(CC)C(N)C). Comparison to the Y polymorph of ROY is included as an example of a dissimilar structure.

	O22	CSP Structure Rank #24	Y
**O22**	0.00	0.18	60.00
**CSP Structure Rank #24**	0.18	0.00	61.09
**Y**	60.00	61.09	0.00

Table S9: Packing similarity calculated using CrystalCMP of the experimentally determined O22 structure, CSP Structure Rank #24, and Y polymorph.

S9. Packing Similarity Analysis of Monomeric ROY Crystal Forms

S9.1 Comparison by Mercury 4.0[S7]

Packing similarity was calculated using the Crystal Packing Similarity feature within Mercury 4.0 using a 15 molecule cluster. Default tolerances for distance (20 %) and angle (20 °) were used. Results are presented as the number of 'molecules in common' (MIC) (i.e. the number of molecules, of the 15-molecule cluster examined, which are judged to achieve a successful overlay within the selected tolerances) and the root mean square deviation (RMSD) in angstroms for the molecules in common.

	Solvate		Y		ON		R		YN		Y04		ORP		R18		Y19		O22	
	MIC	RMSD	MIC	RMSD	MIC	RMSD	MIC	RMSD	MIC	RMSD	MIC	RMSD	MIC	RMSD	MIC	RMSD	MIC	RMSD	MIC	RMSD
Solvate	15	0	1	0.723	3	0.073	1	0.382	1	0.757	1	0.319	3	0.678	1	0.747	8	1	1	0.075
Y	1	0.723	15	0	1	0.736	3	1.073	3	0.12	3	0.493	1	0.589	1	0.474	2	0.823	1	0.734
ON	3	0.073	1	0.736	15	0	1	0.368	1	0.771	2	0.612	1	0.255	3	0.865	4	0.451	1	0.034
R	1	0.382	3	1.073	1	0.368	15	0	1	1.054	3	0.766	5	0.488	1	0.651	2	0.823	2	0.398
YN	1	0.757	3	0.12	1	0.771	1	1.054	15	0	4	0.677	1	0.589	1	1.425	1	0.669	1	0.77
Y04	1	0.319	3	0.493	2	0.612	3	0.766	4	0.677	15	0	1	0.589	1	1.035	1	0.238	3	0.543
ORP	3	0.678	1	0.589	1	0.255	5	0.488	1	0.589	1	0.589	15	0	1	0.474	2	0.666	2	0.4
R18	1	0.747	1	0.474	3	0.865	1	0.651	1	1.425	1	1.035	1	0.474	15	0	2	0.844	1	0.714
Y19	8	1	2	0.823	4	0.451	2	0.823	1	0.238	1	0.238	2	0.666	2	0.844	15	0	1	0.129
O22	1	0.075	1	0.734	1	0.034	2	0.398	1	0.77	3	0.543	2	0.4	1	0.714	1	0.129	15	0

Table S10: Packing similarity using Mercury 4.0 for ROY crystal forms observed via ENaCt. MIC = molecules in common, RMSD = the root mean square deviation of the molecules in common.

S9.2 Comparison by CrystalCMP[S8]

Packing similarity was calculated using the packing similarity feature to compare a 15 molecule cluster in CrystalCMP, using the smiles string (N(C1SCCC1C)C(CC)C(N)C) to compare the structures.

Packing similarity is reported as a single numerical value obtained *via* the following expression:

$$Ps_{a,b} = D_c + wA_d$$

Equation S1: Molecular similarity calculation carried by CrystalCMP. D_c = average centroid-centroid displacement, A_d = average angle between overlapped molecules, w is the weighting of average angle values. Default weighting (w = 2.77) was used.

	Solvate	Y	ON	R	YN	Y04	ORP	R18	Y19	O22
Solvate	0.00	47.74	30.01	46.06	54.02	58.68	31.16	58.81	3.06	54.85
Y	47.74	0.00	24.96	62.37	57.82	57.99	67.42	52.94	31.39	61.09
ON	30.01	24.96	0.00	52.39	64.30	53.17	50.80	59.45	32.48	51.92
R	46.06	62.37	52.39	0.00	45.12	41.28	23.07	46.15	29.31	53.15
YN	54.02	57.82	64.30	45.12	0.00	50.78	36.94	48.55	47.90	53.56
Y04	58.68	57.99	53.17	41.28	50.78	0.00	55.00	45.94	49.30	41.25
ORP	31.16	67.42	50.80	23.07	36.94	55.00	0.00	47.80	21.15	51.62
R18	58.81	52.94	59.45	46.15	48.55	45.94	47.80	0.00	52.52	49.62
Y19	3.06	31.39	32.48	29.31	47.90	49.30	21.15	52.52	0.00	46.88
O22	54.85	61.09	51.92	53.15	53.56	41.25	51.62	49.62	46.88	0.00

Table S11: Packing similarity calculation results using CrystalCMP to compare structures of ROY using the smiles string (N(C1SCCC1C)C(CC)C(N)C).

S10. O22 Crystal Structure Analysis

S10.1 Packing Diagrams of O22

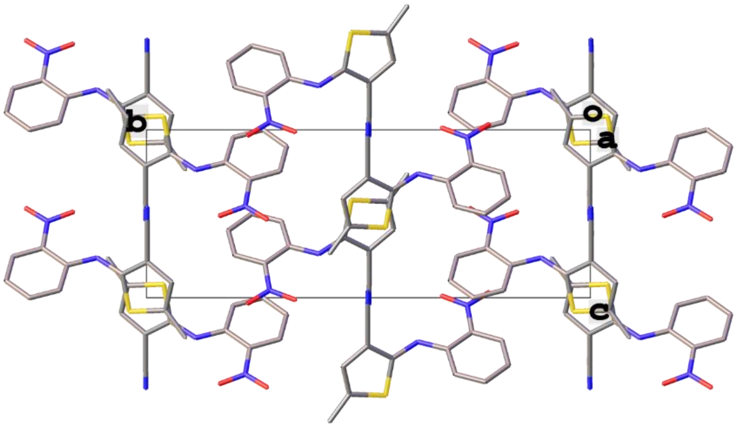

Figure S9: Packing diagram of the O22 polymorph of ROY viewed down the *a*-axis. Hydrogen atoms removed for clarity.

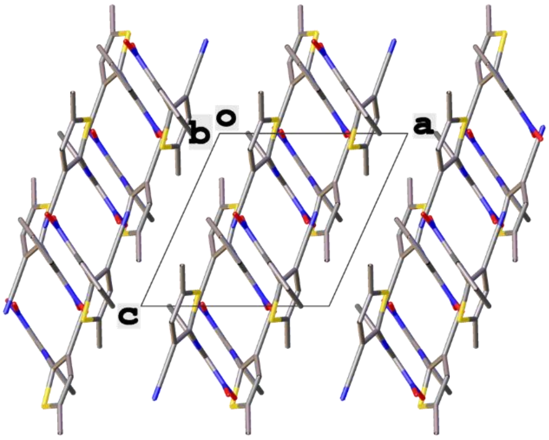

Figure S10: Packing diagram of the O22 polymorph of ROY viewed down the *b*-axis. Atomic displacement ellipsoids and hydrogen atoms removed for clarity.

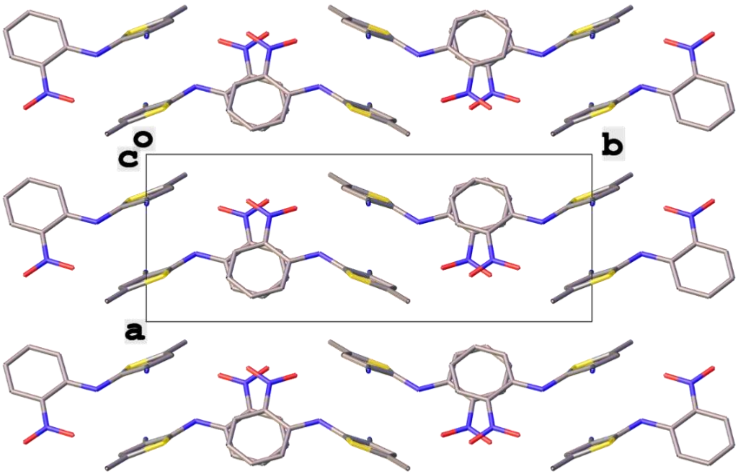

Figure S11: Packing diagram of the O22 polymorph of ROY viewed down the *c*-axis. Atomic displacement ellipsoids and hydrogen atoms removed for clarity.

S10.2 Hirshfeld Surfaces of O22

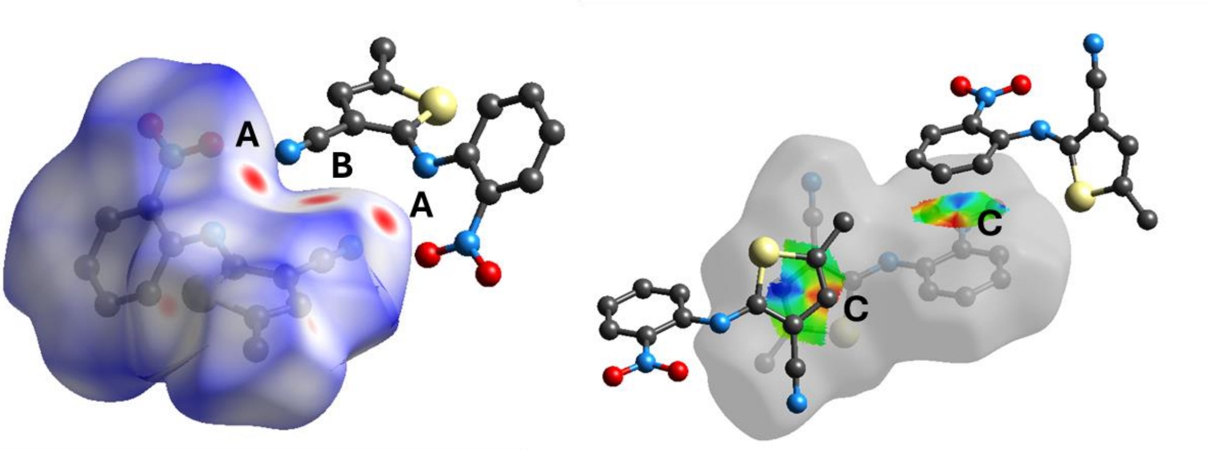

Figure S12: Hirshfeld surface of d_{norm} (left) with NH to nitrile hydrogen bonds (A) and nitrile to nitrile pi-pi interactions (B) highlighted. Hirshfeld suface of shape index (right) with aromatic pi-pi interactions (C) highlighted.

S10.3 Overlay of Experimental O22 Structure with CSP Structure Rank #24

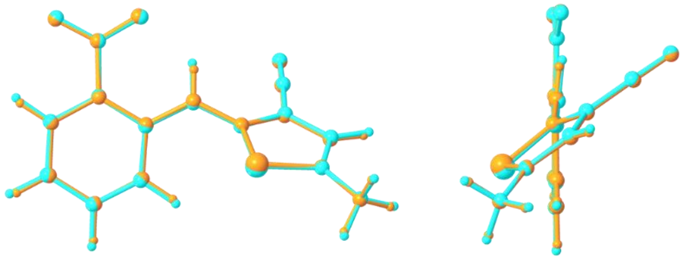

Figure S13: Overlay of ROY molecules within experimentally determined O22 structure (orange) and the CSP Structure Rank #24 (blue).

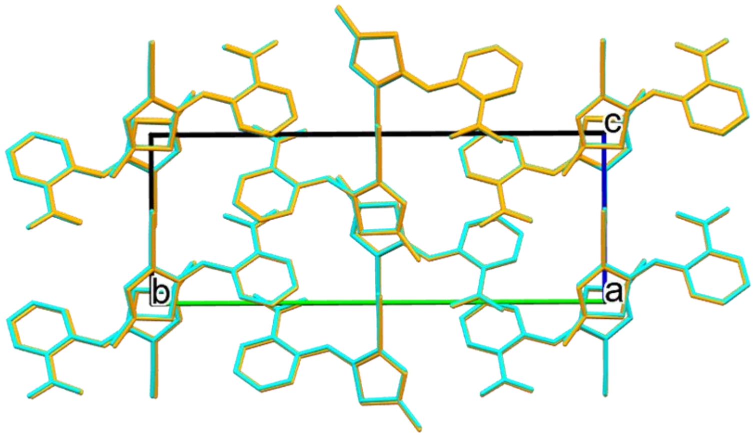

Figure S14: Packing overlay of the experimentally determined O22 structure (orange) and the CSP Structure Rank #24 (blue) as viewed down the *a*-axis. Hydrogen atoms removed for clarity.

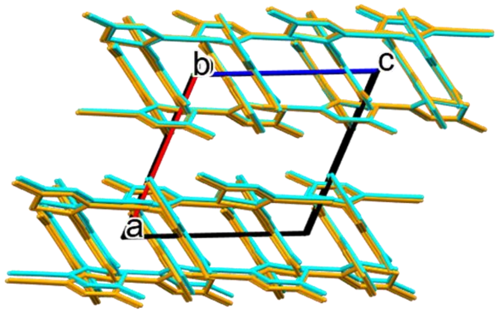

Figure S15: Packing overlay of the experimentally determined O22 structure (orange) and the CSP Structure Rank #24 (blue) as viewed down the *b*-axis. Hydrogen atoms removed for clarity.

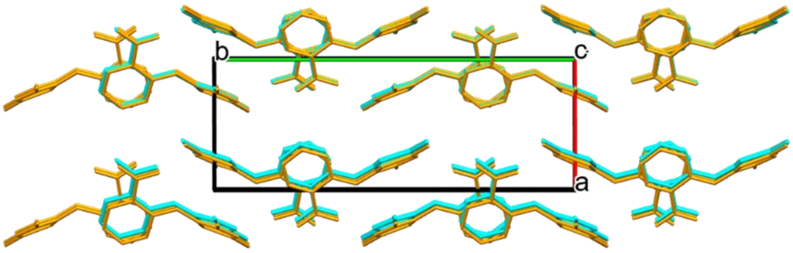

Figure S16: Packing overlay of the experimentally determined O22 structure (orange) and the CSP Structure Rank #24 (blue) as viewed down the *c*-axis. Hydrogen atoms removed for clarity.

S11. ROY·methyl anthranilate solvate Crystal Structure Analysis

S11.1 Packing Diagrams of ROY·methyl anthranilate solvate

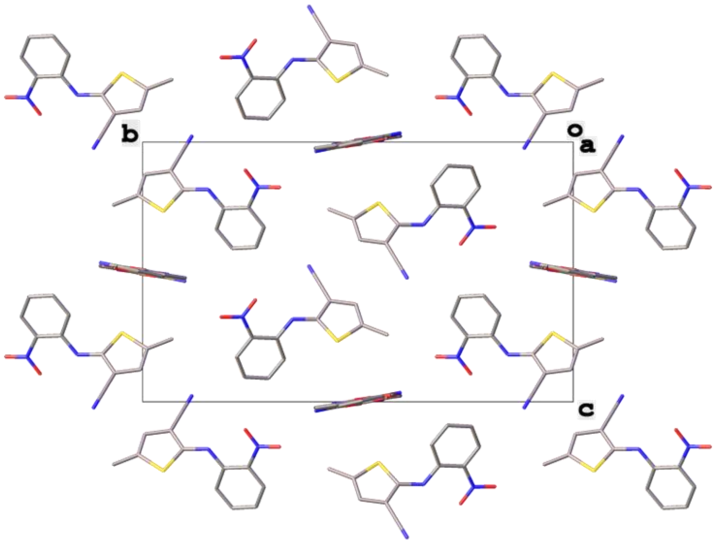

Figure S17: Packing diagram of the ROY·methyl anthranilate solvate as viewed down the *a*-axis. Hydrogen atoms removed for clarity.

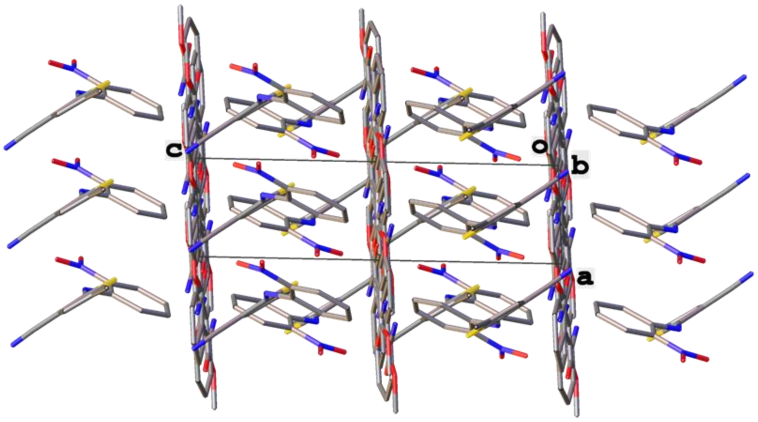

Figure S18: Packing diagram of the ROY·methyl anthranilate solvate as viewed down the *b*-axis. Hydrogen atoms removed for clarity.

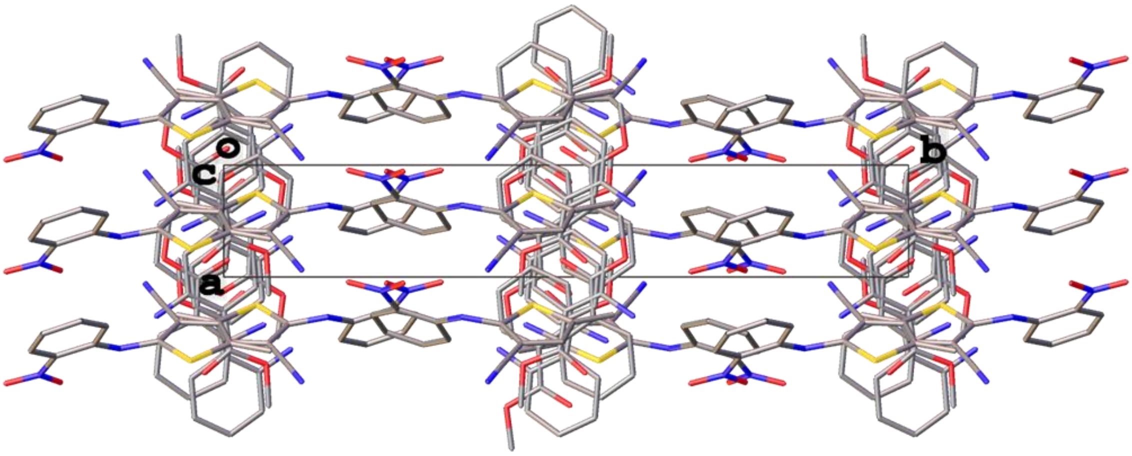

Figure S19: Packing diagram of the ROY·methyl anthranilate solvate as viewed down the *c*-axis. Hydrogen atoms removed for clarity.

S11.2 Hirshfeld Surface of ROY·methyl anthranilate solvate

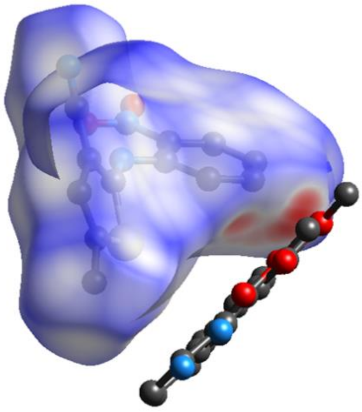

Figure S20: Hirshfeld surface of d_{norm} for a ROY molecule in the ROY·methyl anthranilate solvate, showing the close contacts between the solvent channel and the ROY molecule.

S11.3 Overlay of Experimental ROY·methyl anthranilate solvate Structure with Y19

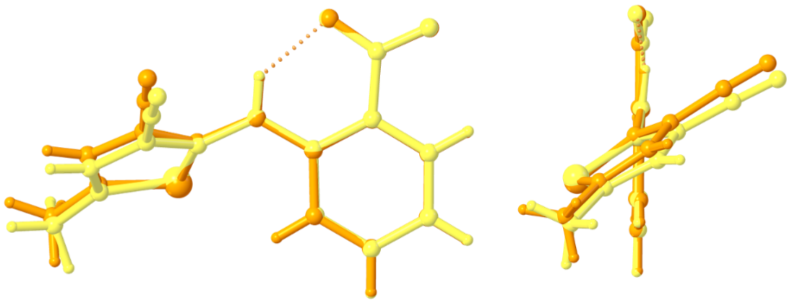

Figure S21: Overlay of ROY molecules in the ROY·methyl anthranilate solvate (orange) and Y19 (yellow).

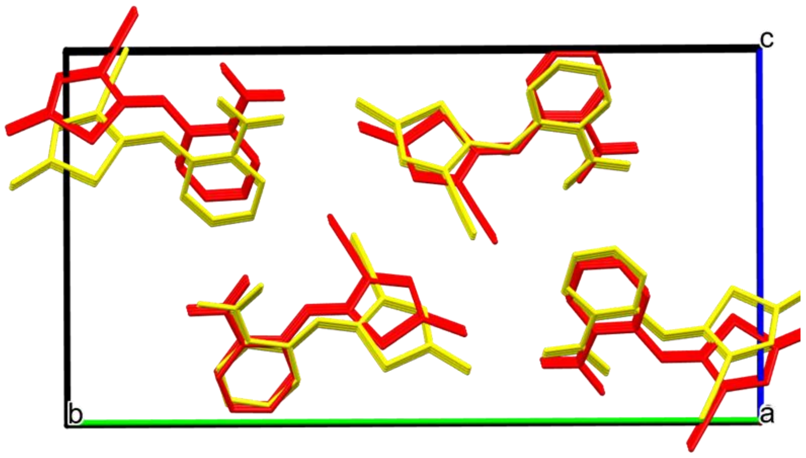

Figure S22: Packing overlay of ROY·methyl anthranilate solvate (red) and Y19 ROY (yellow), as viewed down the *a*-axis. Hydrogen atoms removed for clarity.

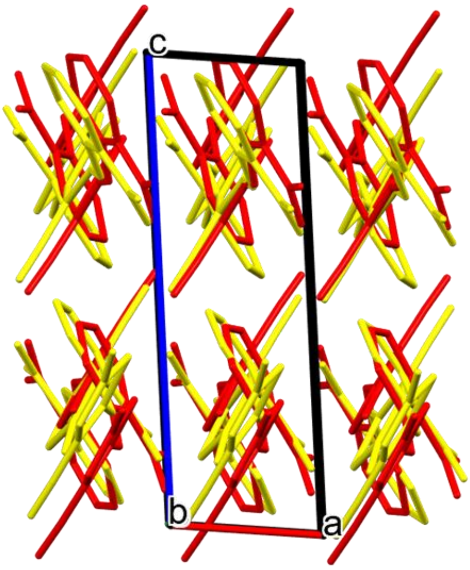

Figure S23: Packing overlay of ROY·methyl anthranilate solvate (red) and Y19 ROY (yellow), as viewed down the *b*-axis. Hydrogen atoms removed for clarity.

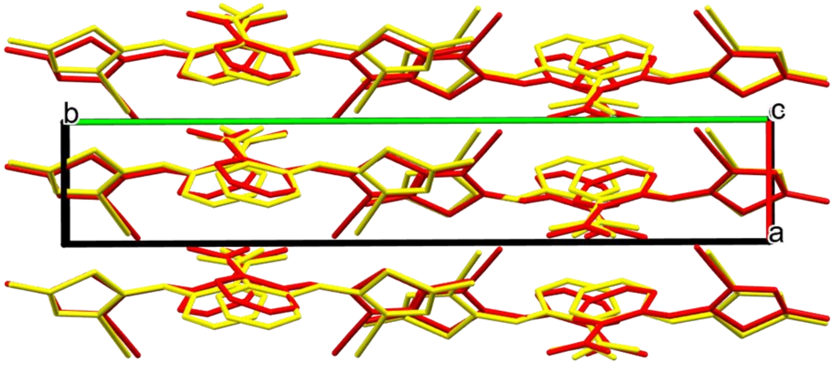

Figure S24: Packing overlay of ROY·methyl anthranilate solvate (red) and Y19 ROY (yellow), as viewed down the *c*-axis. Hydrogen atoms removed for clarity.

S12. ROY Dimer Crystal Structure Analysis

S12.1 Packing Diagrams of ROY dimer

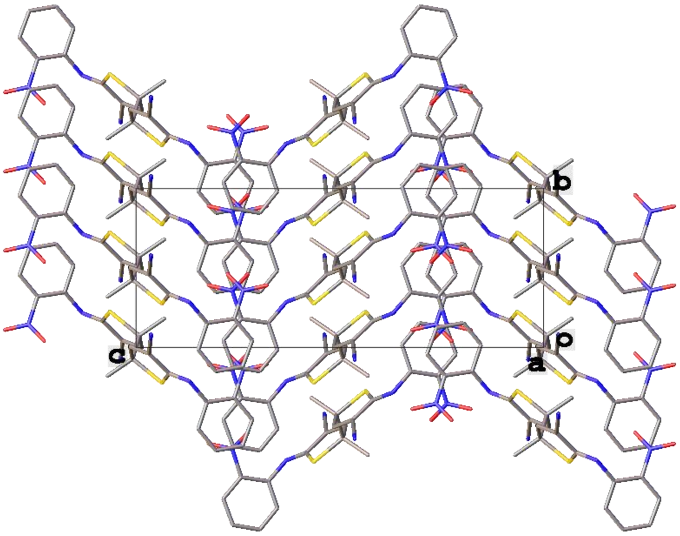

Figure S25: Packing diagram of ROY dimer as viewed down the *a*-axis. Hydrogen atoms removed for clarity.

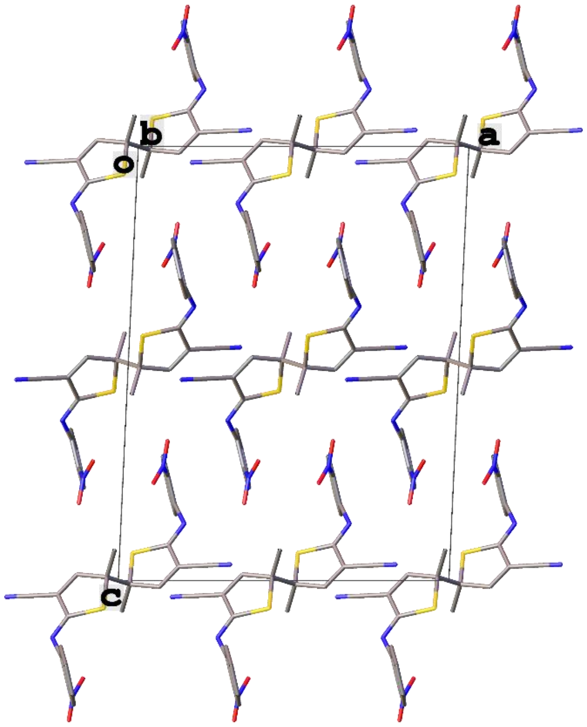

Figure S26: Packing diagram of ROY dimer as viewed down the *b*-axis. Hydrogen atoms removed for clarity.

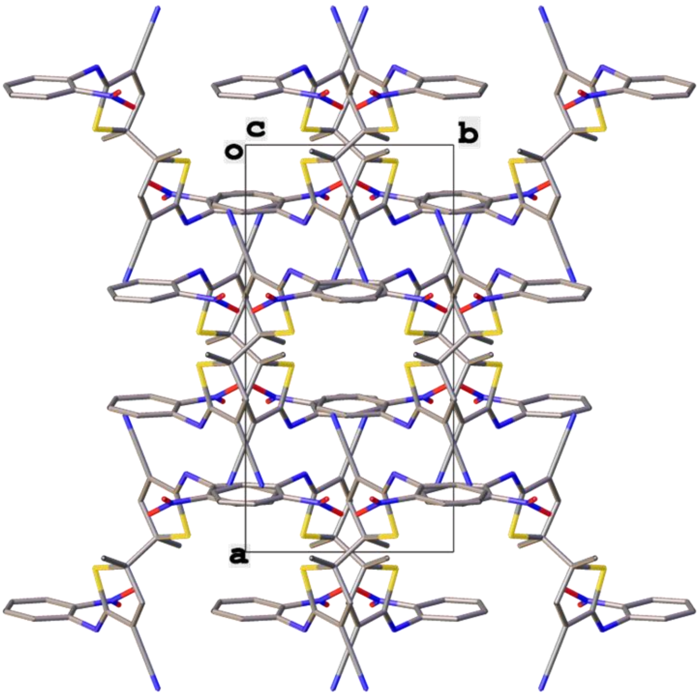

Figure S27: Packing diagram of ROY dimer as viewed down the *c*-axis. Hydrogen atoms removed for clarity.

S12.2 Hirshfeld surfaces of ROY dimer

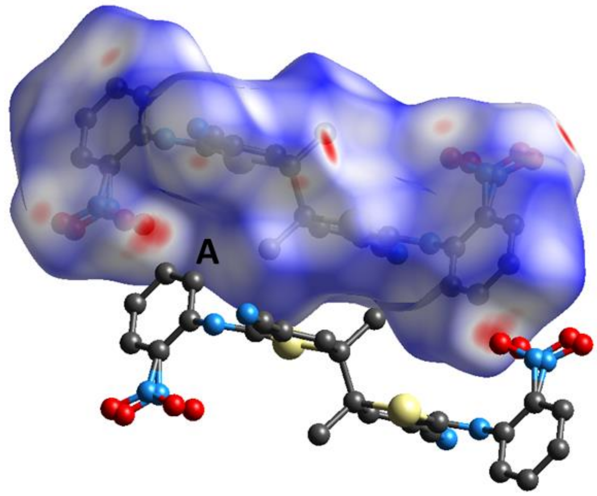

Figure S28: Hirshfeld surface of d_{norm} of ROY dimer with phenyl C-H to nitro N-O close contacts highlighted (A).

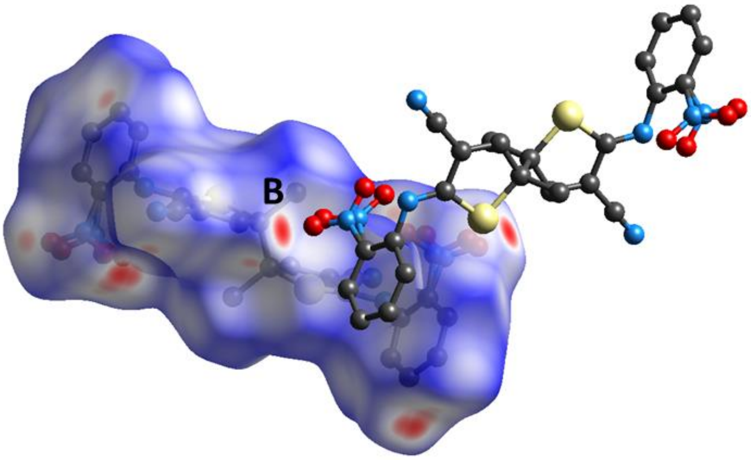

Figure S29: Hirshfeld surface of d_{norm} of ROY dimer with thiophene C-H to nitro N-O close contacts highlighted (B).

S13. Purity Analysis of ROY using in ENaCt Experiments

S13.1 ^{1}H and ^{13}C NMR Analysis of ROY used in ENaCt Experiments

^{1}H and ^{13}C NMR was undertaken to look for the presence of ROY dimer as an impurity in the sample used in ENaCt experiments. No impurities were observable by NMR.

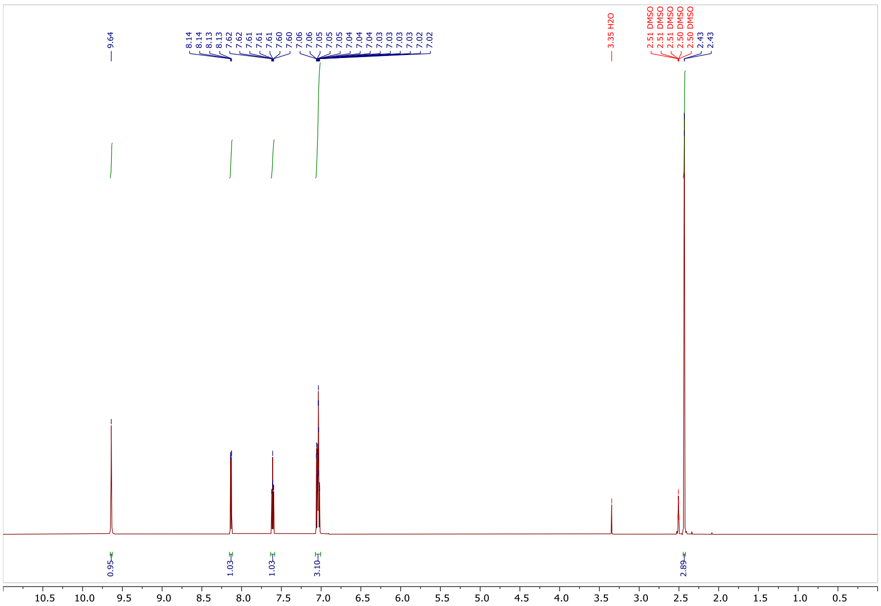

Figure S30: ^{1}H NMR spectrum of the ROY sample used for ENaCt screening (700 MHz, d6-DMSO).

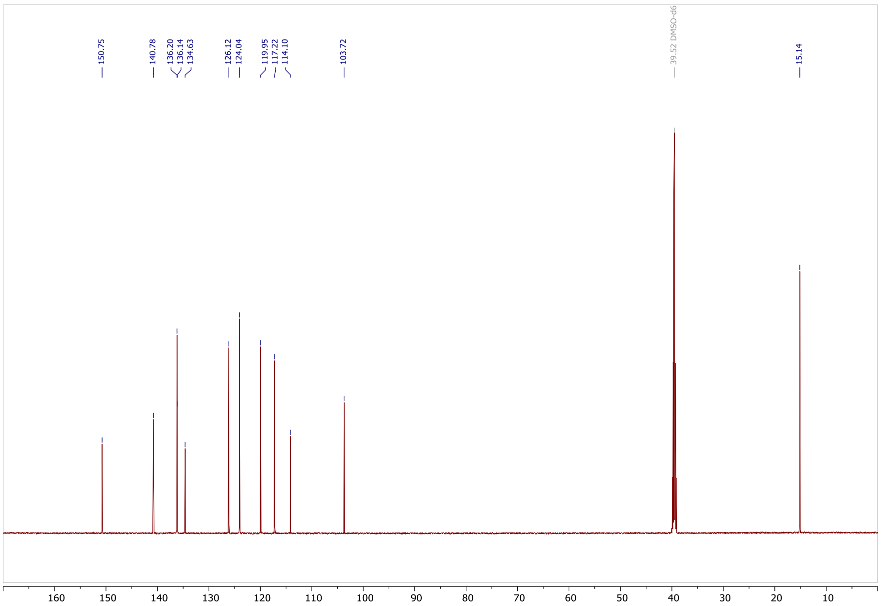

Figure S31: ^{13}C NMR spectrum of the ROY sample used for ENaCt screening (176 MHz, d6-DMSO).

S13.2 HRMS Analysis of ROY used in ENaCt Experiments

High resolution mass spectrometry (Positive mode, Atmospheric Solids Analysis Probe (ASAP), Xevo G2 QTof (EPSRC National Mass Spectrometry Service, University of Swansea)) was undertaken to look for the presence of ROY dimer as an impurity in the sample used in ENaCt experiments.

Peaks at 260.0494 and 242.0390 m/z were observed, corresponding to the [$M+H^+$] and [$M+H-H_2O^+$] ions on ROY. No peaks could be seen at 517.0747 which would correspond to a hypothetical ROY dimer [$M+H^+$], or other likely related molecular ions.

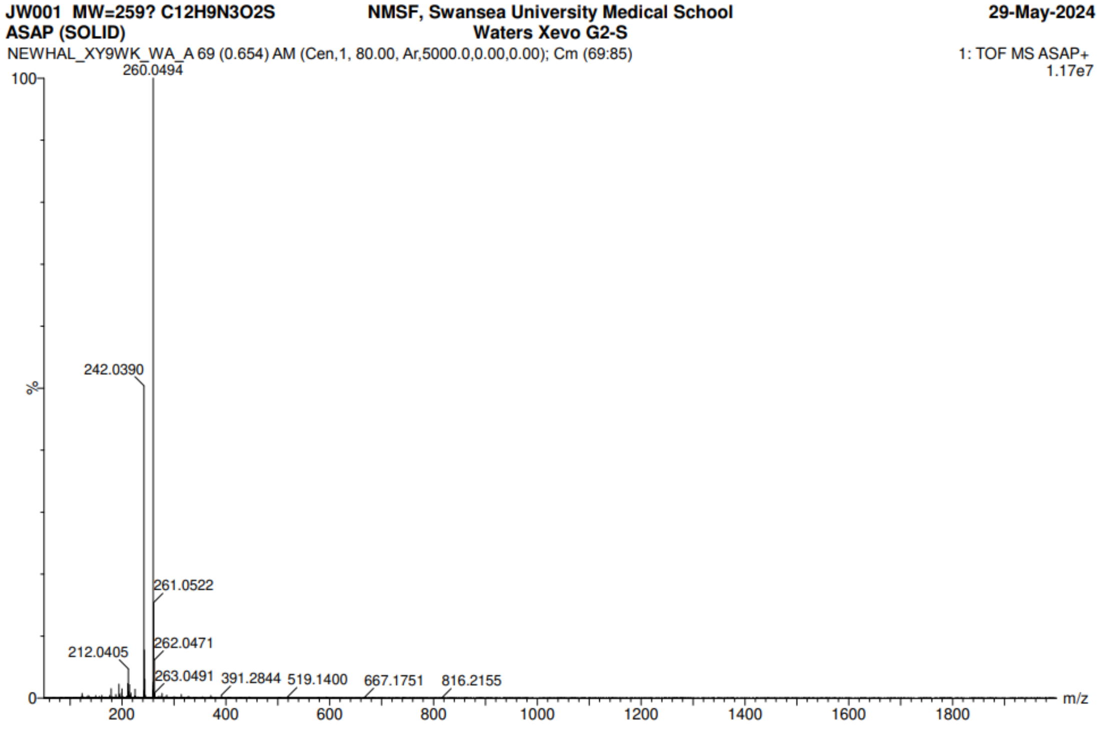

Figure S32: HRMS of ROY used in ENaCt experiments.

S14. Beran CSP Ranking of ROY Polymorphs including O22[S9]

CSP Structure Ranking	Polymorph	Space Group	Relative Calculated Lattice Energy (kJ/mol)
0001	Y	$P2_1/n$	0.000
0002	YT04	$P2_1/n$	0.642
0003	R	$P\bar{1}$	0.825
0004	OP	$P2_1/n$	1.607
0005	YN	$P\bar{1}$	2.313
0006	Y04	P-1	2.417
0007	R05	$P2_1$	2.607
0010	PO13	$P2_1/c$	2.972
0012	ON	$P2_1/c$	3.016
0023	ORP	$Pbca$	4.290
0024	O22	$P2_1/c$	4.371
0032	R18	$P\bar{1}$	4.860
0037	RPL	$Pbca$	5.160
0072	Y19	$P2_1/c$	6.606

Table S12: Relative energies of ROY polymorphs by CSP, adapted from Beran *et al.*. Yellow = polymorphs observed by ENaCt in this study. Green = O22 discovered in this work equated to CSP Structure Rank #24.[S9]

S15. Torsional and Mean Plane Angles of ROY forms

ROY Polymorphs and Forms Reported in this Work			
	τ_{SCNC}	τ_{CNCC}	**Mean Plane Angle, θ**
**Y**	106.15(14)	4.7(2)	107.87(5)
**R**	-19.6(2)	-31.4(3)	45.34(6)
**YN**	104.2(3)	7.4(4)	70.24(1)
**ON**	-54.4(2)	-5.9(2)	53.75(5)
**ORP**	-39.1(2)	-6.5(3)	39.64(5)
**R18a**<br>**R18b**[a]	-10.4(2)<br>-4.8(3)	4.7(3)<br>1.2(3)	6.31(5)<br>4.30(5)
**Y04**	-76.99(19)	-9.2(2)	80.06(6)
**Y19**	-61.7(2)	-5.3(3)	61.95(5)
**O22**	-57.1(3)	-5.3(3)	56.17(7)
**Dimer**	-7.9(6)	-75.5(5)	101.05(16)
**Solvate**	-53.2(3)	-8.7(4)	124.78(9)
**Additional ROY Known (CCDC) and Predicted (CSP) Polymorphs**			
	τ_{SCNC}	τ_{CNCC}	**Plane Angle, θ**
**OP (QAXMEH03)**	46.1(4)	12.4(4)	50.70(9)
**YT04 (QAXMEH12)**	112.84(15)	-10.3(2)	73.55(5)
**R05 a (QAXMEH31)**<br>**R05 b**[a],[c]	-33.997(4)<br>44.881(6)	-19.191(2)<br>11.7984(14)	46.151(5)<br>49.891(6)
**PO13 (QAXMEH52)**	122.057573	12.98974	54.040463
**RPL a**<br>**RPL b**[a],[d]	32.559834<br>23.728137	9.229748<br>10.358947	36.302232<br>29.382006

Table S13. Torsion angles (τSCNC and τCNCC) and mean plane angle θ, between the planes of the aromatic rings calculated using Olex2, from crystal structures from ENaCt grown crystal of each polymorph or from literature data. [a] Z’ = 2, [b] No ESDs reported for PO13 and RPL. [c] R05 structure based on X-ray powder diffraction and crystal structure prediction (CSP),[S10] [d] RPL structure based on RAMAN and crystal structure prediction (CSP).[S11]

S16. Crystallographic Data Tables for Obtained ROY Forms

	Y	R	YN	ON
**Identification code**	NCL_ROY_Y	NCL_ROY_R	NCL_ROY_YN	NCL_ROY_ON
**CCDC Deposition Number**	2381874	2381875	2381876	2381877
**Empirical formula**	$C_{12}H_9N_3O_2S$	$C_{12}H_9N_3O_2S$	$C_{12}H_9N_3O_2S$	$C_{12}H_9N_3O_2S$
**Formula weight**	259.28	259.28	259.28	259.28
**Temperature/K**	150.00	150.00	150.00	150.00
**Crystal system**	monoclinic	triclinic	triclinic	monoclinic
**Space group**	$P2_1/n$	$P\overline{1}$	$P\overline{1}$	$P2_1/c$
**a/Å**	8.4825(8)	7.3451(7)	4.5203(3)	3.8732(2)
**b/Å**	16.1025(15)	7.7009(8)	11.1627(7)	18.5275(7)
**c/Å**	8.5017(8)	11.8830(12)	12.2764(8)	16.3617(6)
**α/°**	90	75.609(2)	108.638(2)	90
**β/°**	91.455(3)	77.512(2)	90.061(2)	92.8730(10)
**γ/°**	90	64.293(2)	92.695(2)	90
**Volume/Å^3**	1160.87(19)	582.07(10)	586.23(7)	1172.65(9)
**Z**	4	2	2	4
**ρ_{calc}g/cm^3**	1.484	1.479	1.469	1.469
**μ/mm^{-1}**	2.475	2.468	2.450	2.450
**F(000)**	536.0	268.0	268.0	536.0
**Crystal size/mm^3**	0.07 × 0.04 × 0.025	0.05 × 0.05 × 0.02	0.09 × 0.002 × 0.002	0.3 × 0.005 × 0.005
**Radiation**	CuKα (λ = 1.54178)	CuKα (λ = 1.54184)	CuKα (λ = 1.54178)	CuKα (λ = 1.54178)
**2Θ range for data collection/°**	10.988 to 144.306	7.74 to 145.158	7.6 to 144.194	7.212 to 144.34
**Index ranges**	-10 ≤ h ≤ 10, -19 ≤ k ≤ 19, -10 ≤ l ≤ 10	-9 ≤ h ≤ 9, -9 ≤ k ≤ 9, -14 ≤ l ≤ 14	-5 ≤ h ≤ 5, -13 ≤ k ≤ 13, -15 ≤ l ≤ 15	-4 ≤ h ≤ 4, -22 ≤ k ≤ 22, -20 ≤ l ≤ 20
**Reflections collected**	18191	20247	11295	15727
**Independent reflections**	2283 [R_{int} = 0.0353, R_{sigma} = 0.0210]	2253 [R_{int} = 0.0245, R_{sigma} = 0.0152]	2248 [R_{int} = 0.0574, R_{sigma} = 0.0418]	2289 [R_{int} = 0.0446, R_{sigma} = 0.0276]
**Data/restraints/parameters**	2283/0/167	2253/0/179	2248/0/169	2289/0/167
**Goodness-of-fit on F^2**	1.159	1.090	1.154	1.105
**Final R indexes [I>=2σ (I)]**	R_1 = 0.0329, wR_2 = 0.0953	R_1 = 0.0295, wR2 = 0.0834	R_1 = 0.0674, wR_2 = 0.1919	R_1 = 0.0369, wR_2 = 0.1035
**Final R indexes [all data]**	R_1 = 0.0337, wR_2 = 0.0961	R_1 = 0.0296, wR2 = 0.0834	R_1 = 0.0736, wR_2 = 0.2035	R_1 = 0.0391, wR_2 = 0.1059
**Largest diff. peak/hole / e Å^{-3}**	0.29/-0.41	0.30/-0.33	0.38/-0.50	0.21/-0.32

	ORP	R18	Y04	Y19
Identification code	NCL_ROY_ORP	NCL_ROY_R18	NCL_ROY_Y04	NCL_ROY_Y19
CCDC Deposition Number	2381878	2381879	2381880	2381881
Empirical formula	$C_{12}H_9N_3O_2S$	$C_{12}H_9N_3O_2S$	$C_{12}H_9N_3O_2S$	$C_{12}H_9N_3O_2S$
Formula weight	259.28	259.28	259.28	259.28
Temperature/K	150.00	149.99(10)	150.00	150.00
Crystal system	orthorhombic	triclinic	triclinic	monoclinic
Space group	P*bca*	$P\overline{1}$	$P\overline{1}$	$P2_1/c$
a/Å	12.9085(2)	8.2352(2)	6.9758(5)	4.06030(10)
b/Å	7.99440(10)	11.6405(3)	8.2772(6)	23.2892(8)
c/Å	22.7861(4)	12.7276(3)	10.3752(8)	12.5584(4)
α/°	90	89.391(2)	97.578(2)	90
β/°	90	73.116(2)	103.257(2)	96.485(3)
γ/°	90	87.380(2)	90.059(2)	90
Volume/$Å^3$	2351.43(6)	1166.27(5)	577.70(7)	1179.94(6)
Z	8	4	2	4
$\rho_{calc}g/cm^3$	1.465	1.477	1.491	1.460
μ/mm^{-1}	2.443	2.463	2.486	2.435
F(000)	1072.0	536.0	268.0	536.0
Crystal size/mm^3	0.3 × 0.1 × 0.01	0.089 × 0.073 × 0.05	0.08 × 0.04 × 0.04	0.3 × 0.01 × 0.01
Radiation	CuKα (λ = 1.54184)	CuKα (λ = 1.54184)	CuKα (λ = 1.54178)	CuKα (λ = 1.54184)
2Θ range for data collection/°	7.76 to 152.59	7.258 to 153.054	8.838 to 133.328	7.592 to 156.69
Index ranges	-16 ≤ h ≤ 16, -5 ≤ k ≤ 9, -27 ≤ l ≤ 25	-10 ≤ h ≤ 10, -14 ≤ k ≤ 14, -15 ≤ l ≤ 12	-8 ≤ h ≤ 8, -9 ≤ k ≤ 9, -12 ≤ l ≤ 12	-3 ≤ h ≤ 4, -29 ≤ k ≤ 28, -15 ≤ l ≤ 15
Reflections collected	8982	12194	16850	7600
Independent reflections	2319 [R_{int} = 0.0307, R_{sigma} = 0.0257]	4522 [R_{int} = 0.0211, R_{sigma} = 0.0255]	2017 [R_{int} = 0.0350, R_{sigma} = 0.0221]	2350 [R_{int} = 0.0353, R_{sigma} = 0.0352]
Data/restraints/parameters	2319/0/168	4522/258/335	2017/0/165	2350/0/168
Goodness-of-fit on F^2	1.055	1.057	1.149	1.061
Final R indexes [I>=2σ (I)]	R_1 = 0.0347, wR_2 = 0.0929	R_1 = 0.0337, wR_2 = 0.0921	R_1 = 0.0363, wR_2 = 0.1013	R_1 = 0.0380, wR_2 = 0.1009
Final R indexes [all data]	R_1 = 0.0397, wR_2 = 0.0971	R_1 = 0.0385, wR_2 = 0.0949	R_1 = 0.0370, wR_2 = 0.1019	R_1 = 0.0437, wR_2 = 0.1053
Largest diff. peak/hole / e $Å^{-3}$	0.21/-0.28	0.34/-0.20	0.23/-0.33	0.32/-0.23

	O22	Dimer	Solvate	
**Identification code**	NCL_ROY_O22	NCL_ROY_DIMER	NCL_ROY_MA_SOLVATE	
**CCDC Deposition Number**	2381882	2381883	2381884	
**Empirical formula**	$C_{12}H_9N_3O_2S$	$C_{24}H_{16}N_6O_4S_2$	$C_{13.91}H_{9.67}N_{3.24}O_{2.48}S$	
**Formula weight**	259.28	516.55	293.83	
**Temperature/K**	150.00	150.00	150.00	
**Crystal system**	monoclinic	monoclinic	monoclinic	
**Space group**	$P2_1/c$	$C2/c$	$P2_1/c$	
**a/Å**	8.0874(5)	15.1969(4)	3.88127(11)	
**b/Å**	19.6111(13)	7.7513(2)	23.7098(7)	
**c/Å**	8.1701(6)	19.9721(5)	14.3056(3)	
**α/°**	90	90	90	
**β/°**	114.455(2)	92.6400(10)	93.145(3)	
**γ/°**	90	90	90	
**Volume/Å^3**	1179.55(14)	2350.13(10)	1314.48(6)	
**Z**	4	4	4	
**ρ_{calc}g/cm^3**	1.460	1.460	1.485	
**μ/mm^{-1}**	2.435	2.444	2.294	
**F(000)**	536.0	1064.0	606.0	
**Crystal size/mm^3**	0.07 × 0.05 × 0.03	0.05 × 0.03 × 0.005	0.585 × 0.012 × 0.011	
**Radiation**	CuKα (λ = 1.54178)	CuKα (λ = 1.54178)	CuKα (λ = 1.54184)	
**2Θ range for data collection/°**	9.018 to 144.874	8.864 to 133.41	7.226 to 156.802	
**Index ranges**	-10 ≤ h ≤ 9, -24 ≤ k ≤ 23, -9 ≤ l ≤ 9	-18 ≤ h ≤ 18, -9 ≤ k ≤ 9, -23 ≤ l ≤ 23	-4 ≤ h ≤ 2, -28 ≤ k ≤ 28, -18 ≤ l ≤ 18	
**Reflections collected**	17485	15623	12454	
**Independent reflections**	2320 [R_{int} = 0.0445, R_{sigma} = 0.0312]	2088 [R_{int} = 0.0566, R_{sigma} = 0.0330]	12454 [R_{int} = 0.0980, R_{sigma} = 0.0344]	
**Data/restraints/parameters**	2320/0/168	2088/231/222	12454/396/213	
**Goodness-of-fit on F^2**	1.112	1.064	1.049	
**Final R indexes [I>=2σ (I)]**	R_1 = 0.0387, wR_2 = 0.1072	R_1 = 0.0665, wR_2 = 0.1804	R_1 = 0.0437, wR_2 = 0.1062	
**Final R indexes [all data]**	R_1 = 0.0408, wR_2 = 0.1092	R_1 = 0.0763, wR_2 = 0.1904	R_1 = 0.0568, wR_2 = 0.1112	
**Largest diff. peak/hole / e Å^{-3}**	0.21/-0.35	0.92/-0.34	0.24/-0.23	

Table S14: Crystallographic Data for ROY Forms.

S17. References

S1. APEX3, SAINT and SADABS, Bruker AXS Inc., Madison, Wisconsin, USA.

S2. CrysAlisPRO, Oxford Diffraction/Agilent Technologies UK Ltd, Yarnton, England.

S3. Cosier, J; Glazer, A. M. A Nitrogen-Gas-Stream Cryostat for General X-ray Diffraction Studies. *Journal of Applied Crystallography* **1986**, *19*, 105–107. https://doi.org/10.1107/S0021889886089835.

S4. Sheldrick, G. M. Crystal Structure Refinement with SHELXL. *Acta Crystallographica* **2015**, *C71*, 3–8. http://doi.org/10.1107/S2053229614024218.

S5. Sheldrick, G. M. SHELXT-Integrated Space-Group and Crystal-Structure Determination. *Acta Crystallographica,* **2015**, *A71*, 3–8. https://doi.org/10.1107/S2053273314026370.5.

S6. Dolomanov, O. V.; Bourhis, L. J.; Gildea, R. J.; Howard, J. A. K.; Puschmann, H. OLEX2: A Complete Structure Solution, Refinement and Analysis Program. *Journal of Applied Crystallography* **2009**, *42*, 339–341. http://doi.org/10.1107/S0021889808042726.

S7. Macrae, C. F.; Sovago, I.; Cottrell, S. J.; Galek, P. T. A.; McCabe, P.; Pidcock, E.; Platings, M.; Shields, G. P.; Stevens, J. S.; Towler M.; Wood, P. A. Mercury 4.0: from visualization to analysis, design and prediction, *Journal of Applied Crystallography*, **2020**, *53*, 226–235. http://doi.org/10.1107/S1600576719014092.

S8. Rohliček, J.; Skořepova, E.; CrystalCMP: automatic comparison of molecular structures *Journal of Applied Crystallography*, **2020**, *53*, 841-847. http://doi.org/10.1107/S1600576720003787.

S9. Beran, G. J. O.; Sugden, I. J.; Greenwell, C.; Bowskill, D. H.; Pantelides C. C.; Adjiman, C. S. How many more polymorphs of ROY remain undiscovered. *Chemical Science*, **2022**, *13*, 1288–1297. http://doi.org/10.1039/D1SC06074K

S10. Tan, M.; Shtukenberg, A.; Xu, W.; Dooryhee, E.; Nichols, S.; Ward, M.; Kahr, B.; Zhu, Q. ROY revisited, again: the eighth solved structure. *Faraday Discussions*, **2018**, *211*, 477–491. http://doi.org/10.1039/C8FD00039E

S11. Nyman, J.; Yu L.; Reutzel-Edens, S. M. Accuracy and reproducibility in crystal structure prediction: the curious case of ROY. *CrystEngComm*, **2019**, *21*, 2080–2088. http://doi.org/10.1039/C8CE01902A
